# Longissimus Dorsi Muscle Transcriptomic Analysis of Simmental and Chinese Native Cattle Differing in Meat Quality

**DOI:** 10.3389/fvets.2020.601064

**Published:** 2020-12-15

**Authors:** Xiangren Meng, Ziwu Gao, Yusheng Liang, Chenglong Zhang, Zhi Chen, Yongjiang Mao, Bizhi Huang, Kaixing Kui, Zhangping Yang

**Affiliations:** ^1^School of Tourism and Culinary Science, Yangzhou University, Yangzhou, China; ^2^Jiangsu Huai-yang Cuisine Engineering Center, Yangzhou University, Yangzhou, China; ^3^Mammalian Nutrition Physiology Genomics, Department of Animal Sciences and Division of Nutritional Sciences, University of Illinois, Urbana, IL, United States; ^4^College of Animal Science and Technology, Yangzhou University, Yangzhou, China; ^5^Academy of Grassland and Animal Science, Yunnan, China

**Keywords:** Chinese native cattle, Simmental, ripening, meat quality, cooked degree

## Abstract

With the rapid development of economy, the demand for beef, with regard to quantity and quality, by consumers has been increasing in China. Chinese native cattle are characterized by their abundant genetic resources, unique origins, large breeding stocks, and robust environmental adaptability. Thus, to explore the genetic mechanisms on regulating meat quality in Chinese native cattle is of great importance to satisfy increased requirements for beef production. In this study, we investigated three breeds of cattle, namely Yunling, Wenshan, and Simmental, at the age of 12 months. Animals were classified into three groups (*n* = 5/breed). Growth traits including body weight and body size and plasma hormone levels were measured. Body weight of Wenshan cattle was significantly lower than that of Yunling and Simmental cattle (*P* < 0.05). Again, body size indexes, such as withers height, body slanting length, chest circumference, and hip and rump length, were significantly lower in Wenshan cattle than those in Yunling and Simmental cattle (*P* < 0.05). However, there were no significant differences in those indexes between Yunling and Simmental cattle (*P* > 0.05). Cattle were slaughtered at the age of 18 months and then meat color, pH, pressing losses, muscle tenderness, and cooking losses were measured at 0, 1, 2, 3, 5, and 7 days. Data revealed differences in meat quality among the three breeds analyzed. Based on transcriptomic sequencing and bioinformatic analysis, we observed 3,198 differentially expressed genes related to meat quality, of which 1,750 genes were upregulated. Moreover, we found two important signaling pathways closely linked to meat quality, namely adipocytokine signaling pathway [e.g., *Leptin receptor* (*LEPR*)] and protein processing in the endoplasmic reticulum [e.g., *signal transducer and activator of transcription 3* (*STAT3*), *heat shock protein* (*HSPA12A*), and *calpain 1* (*CAPN1*)]. The results of transcripts were further verified by qRT-PCR. Using correlation analysis between gene expression levels and shear force, we also identified two functional genes (e.g., *HSPA12A* and *CAPN1*) associated with meat quality. Overall, this study provides new sights into novel targets and underlying mechanisms to modulate meat quality in Chinese native cattle.

## Introduction

With the development of economy, the dietary structure of Chinese consumers has been altered. During this process, beef has gained prominence in China's meat market (1). The rapid increase in consumers' demand for livestock products, especially beef, has led to an insufficient supply of beef (2–4). Therefore, the development of native beef cattle production is of great significance for promoting the improvement of dietary structure and people's living standards in China. According to the statistics of beef cattle industrialization technology system, the annual demand and supply gap of beef in China is approximately 1.6 million tons. China's native cattle genetic resources are rich, with a large number, various breeds, strong feed tolerance, and stress resistance capacity. However, compared with widely used breeds, native breeds usually have smaller size and lower growth rate, along with poorer meat production performance, which hinder the development of local beef production in China. In this study, local Chinese breeds of Yunling cattle and Wenshan cattle were investigated, in comparison with the conventional breed, Simmental cattle. First, body weight, body size, and growth hormone levels in plasma were evaluated. Second, meat quality indicators of the longissimus dorsi muscle were measured including meat color, pH, pressing losses, tenderness, and cooking losses. Lastly, cooking differences using different cooked systems were measured to reveal the effect of tenderness on steak quality under different acidic conditions. Overall, our study potentially contributes to promoting the development of native beef cattle industry in China.

To date, genomic sequencing in most livestock has been completed (5, 6). This study may help elucidate molecular mechanisms governing meat quality in specific cattle breeds. Previous studies have used transcriptomic sequencing to evaluate molecular mechanisms controlling species-specific traits. Huang et al. obtained 19,043 known genes and 1,785 novel genes in bovine early embryos through transcriptomic sequencing as a reproduction-related database (7). Wickramasinghe et al. used Holstein cow somatic cells as research objects and analyzed them by transcriptomic sequencing to determine gene expression across different lactation stages (8). Of particular interest, there were 16,892 differentially expressed genes during prelactation, 19,094 differentially expressed genes during mid-lactation, and 18,070 differentially expressed genes during late lactation. Pathways include whey protein and casein metabolism and fat metabolism. However, the former mainly displayed during prelactation. Zhang et al. screened 1,300 differentially expressed genes in muscle tissue between small-tailed Han sheep and Dubo sheep *via* transcriptomic sequencing (9). He et al. analyzed the longissimus muscle in Qinchuan cattle at different periods by RNA-Seq technology. A total of 6,800 differentially expressed genes were screened using the *P* < 0.05 principle, of which 1,893 genes were upregulated in the fetal calf group (10). The reliability of the sequencing results was verified by qPCR, and 47 differentially expressed genes were selected from the sequencing results. Quantitative PCR detection revealed that the expression levels of 47 genes in the latissimus dorsal muscle were in line with the sequencing results. Therefore, RNA-Seq transcriptomic sequencing serves as an important strategy for exploring genetic potentials in livestock. In the current study, samples with significant differences in meat quality were used for RNA-Seq transcriptomic sequencing to screen and identify pathways and target genes associated with growth and development in Chinese native cattle breeds.

## Materials and Methods

### Experimental Animals

Animals (*n* = 5/breed) selected from 1-year-old Yunling cattle, Wenshan cattle, and Simmental bulls were raised under the same breeding environment. After growth and development measurement and the determination of meat quality, three Yunling cattle and three Simmental cattle were selected. The cattle were fed twice a day with adequate water and the manure was cleaned daily. Approximately 350 g of longissimus dorsi (LD) samples between the 12th to 13th ribs of each cattle were taken. Longissimus dorsi muscle tissue was obtained and immediately placed into liquid nitrogen and subsequently brought to the laboratory for storage at −70°C.

### Weight and Body Size

Growth traits were measured every month. Body weight: body weight was measured using a land scale before morning feeding; body height: the vertical distance from the highest point of the bun nail to the ground; body slant length: the distance from the shoulder end to the ischial end; body straight length: the horizontal distance from the shoulder end to the vertical line of the posterior edge of the ischial end; chest circumference: the circumference around the chest by the vertical axis of the posterior angle of the scapula; tube circumference: the circumference at the thinnest point of the tube bone, generally in the tibia of the left front leg measure from the bottom to the top third.

### Hormone Determination

Blood was collected from Yunling cattle, Wenshan cattle, and Simmental cattle from the jugular vein and stored at −20°C. After centrifugation, plasma was collected and determined by enzyme-linked immunosorbent assay (ELISA) kits (Promega Corp, Beijing, China) for thyroid-stimulating hormone (TSH), triiodothyronine (T3), thyroxine (T4), growth hormone (GH), insulin, and insulin-like growth factor-1 (IGF-1). The absorbance (OD value) was measured at 450 nm using an enzyme standard instrument, and concentrations of hormones were calculated by standard curves.

### Chemical Composition

Approximately 350 g of LD samples between the 12th to 13th ribs of each cattle were taken and experienced a 7-day aging under vacuum conditions at 4°C to evaluate meat quality (11). Sampling was performed on days 0, 1, 2, 3, 5, and 7 (12). The composition of beef was measured using a Foss Lab Meat/Food Composition fast analyzer (FOSS Ltd., Hillerød, Denmark). The 200-g samples on days 0, 3, and 7 were separated from the LD sample and extended into the analysis plate. The results were the average of 16 technical replicates per sample. The equipment was calibrated to the Soxhlet method (13). Protein, collagen, moisture, and fat content were recorded.

### Color Measurement

Samples collected on days 0, 1, 2, 3, 5, and 7 were exposed to the air for 30 min (blooming) at 4°C to measure their color, and color was measured with a Chroma Meter CR-400 colorimeter (Konica Minolta, Inc., Tokyo, Japan) based on luminance (L^*^), redness (a^*^), and yellowness (b^*^) in the CIELab color space. The color values were the average of six scans for each LD. The C^*^ and H^*^ values were calculated from the a^*^ and b^*^ values using the following respective formulas: C^*^ = (a^*^2 + b^*^2)^0.5^ and H^*^ = arctan (b^*^/a^*^) (14, 15).

### pH Value

The pH of each sample collected on days 0, 1, 2, 3, 5, and 7 was measured by a pH meter (Eutech Instruments, pH Spear, USA) (16). Calibration of the pH electrode was performed with standardized buffers (pH 4.0 and 7.0). Each LD sample was measured three times.

### Cooking Loss and Shear Force

The cooking loss rate was measured by the direct weight method (17). Thirty grams of LD was taken from the LD samples collected on days 0, 1, 2, 3, 5, and 7. Next, it was wrapped and sealed in a cooking bag. Then, it was cooked in a water bath at 80°C. When the central temperature reached 70°C, the sample was unwrapped and the surface moisture was dried. The cooking loss rate was calculated according to the formula: cooking loss (%) = (Wc1–Wc2)/Wc1 × 100%. Wc1 represented the weight of the sample before cooking and Wc2 represented the weight of the sample after cooking. Then, to measure the Warner–Bratzler shear force (WBSF), approximately 3-cm-thick LD sample was removed from the cooked LD sample, and the WBSF was measured using the method of Luo et al. (18). Each LD sample was measured three times.

### Water Loss Rate (Muscle Tenderness)

A 2 × 2 × 1 cm meat piece was cut from the LD samples collected on days 0, 1, 2, 3, 5, and 7. Then, a pressure of 343 N (35 kg) was applied to it and maintained for 5 min. The water loss rate was calculated according to the following formula: water loss rate (%) = (Ww1–Ww2)/Ww1 × 100%. Ww1 represented the weight of the sample before water loss and Ww2 represented the weight of the sample after water loss. Each LD sample was measured three times.

### Transcriptomic Sequencing

Total RNA was extracted from tissue using the TRIzol reagent (catalog number: 15596026, Invitrogen, Carlsbad, CA, USA). The RNA integrity number (RIN) method was used to detect RNA quality, and RIN values are above 7.1. Only good-quality RNA was used for this experimental study. Total RNA was extracted from muscle tissue, and then DNA was digested with DNase (Tiangen, Shanghai, China). Subsequently, mRNA was broken into short segments by adding an interruption reagent, and a strand of cDNA was synthesized with six base random primers using the interrupted mRNA as a template. Double-strand cDNA was purified using a commercial kit (Promega Corp, Beijing, China); the purified double-strand cDNA was repaired, combined with an A-tail, and connected with a sequencing connector. The constructed library was qualified by an Agilent 2100 Bioanalyzer and sequenced by Illumina HiSeq 2500 (19).

#### Gene Expression Abundance

For transcriptomic sequencing analysis, we estimated gene expression levels by counting the sequencing sequences (reads) located in the genomic region or gene exons. In addition to the true expression levels of genes, read count is also positively related to gene length and sequencing depth.

#### Analysis of Differential Gene Expression

RNA-Seq data were used to compare and analyze whether there was differential expression of the same unigene in two samples. Two criteria were used to determine differentially expressed genes: one is fold change and the other is *P*-value or false discovery rate (FDR) (padjust). The calculation method of the FDR value should first calculate the *P*-value for each unigene and then use the FDR error control method to test and correct multiple assumptions for the *P*-value.

#### Cluster Analysis

Differentially expressed genes were clustered by unsupervised hierarchical clustering. Based on calculating the distance between two samples, a distance matrix is constructed. The samples can appear in the same cluster through clustering, and genes in the same cluster may have similar biological functions.

#### Gene Ontology and Enrichment Analysis of Kyoto Encyclopedia of Genes and Genomes

We analyzed differentially expressed genes using gene ontology (GO) and described their functions. The number of differential genes in each GO item was counted, and the significance of differential gene enrichment in each GO item was calculated by the hypergeometric distribution test. According to the results of GO analysis and biological significance, we selected genes for a follow-up study.

The Kyoto Encyclopedia of Genes and Genomes (KEGG) is mainly a public database on pathways. We used the KEGG database to analyze differentially expressed genes and used the hypergeometric distribution test to calculate the significance of differentially expressed gene enrichment in each pathway entry. Pathway analysis is helpful to the experimental results. According to the pathway analysis, we found the pathway items that enrich differential genes and determine which molecular pathway alteration might be related to the differentially expressed genes in different samples.

### Fluorescent Quantitative PCR Detection

According to the results of transcriptomic sequencing, five important differentially expressed genes (*LEPR, STAT3, leptin, HSPA12A*, and *CAPN1*) were selected. Premier 6.0 was used to design primers for the selected genes (Table 1) (Tiangen, Shanghai, China). β-Actin was used as an internal reference gene. The reaction system was 20 μL: 1 μL of cDNA, 0.4 μL (10 μmol/L) of upstream and downstream primers, 0.4 μL of Rox reference dye II (50x), 10 μL of SYBR green real-time PCR Master Mix (2x), and 7.8 μL of ddH_2_O (Tiangen, Shanghai, China). Real-time PCR running conditions are as follows: predenaturation at 95°C for 15, 5 s at 95°C, and 34 s at 60°C, 40 cycles. The dissolution curve was analyzed after amplification. Each sample was measured in triplicate, and the average value was taken. Relative quantitative results were calculated by the 2^−ΔΔCT^ method (20).

**Table 1:** Information of primer sequences.

**Gene**	**Accession number**	**Primer sequence**	**Length (bp)**
*Leptin*	XM_010804453.2	F: 5′-CAATGACATCTCACACACGCAG-3′	116
		R: 5′-TCGCCAATGTCTGGTCCATC-3′	
*LEPR*	NM_001206441.1	F: 5′-CTGTGCCACCTTTCTCGTGG-3′	70
		R: 5′-TTGGAAAGAAGGACCCTCTGC-3′	
*STAT3*	NM_001012671.2	F: 5′-AAGGGATTCCCAAGGATGCC-3′	75
		R: 5′-AATTTGAATGCAGTGGCCAGG-3′	
*HSPA1A*	NM_203322.2	F: 5′-AGCCTGGAGAGAGCTGATAAAA-3′	118
		R: 5′-CCCACAGGATCAACGACGTA-3′	
*CAPN1*	NM_174259.2	F: 5′-GCTGACCATGTTTGCGTGAG-3′	190
		R: 5′-AGAGCAAATGAAACACGGCG-3′	
*β-actin*	XM_003124280.3	F: 5′-TGGCGCCCAGCACGATGAAG-3′	149
		R: 5′-GATGGAGGGGCCGGACTCGT-3′	

### Statistical Analysis

Statistical analysis was performed by two-way analysis of variance (ANOVA) using the Tukey–Kramer adjusted generalized linear model (GLM) procedures of Statistical Analysis Software (SAS) 9.4 (SAS Institute, Cary, NC, USA). *P* < 0.05 indicates significant differences, and *P* < 0.01 indicates highly significant differences (21).

## Results

### Three Cattle Breeds' Growth Performance and Carcass Traits

**Figure 1:**
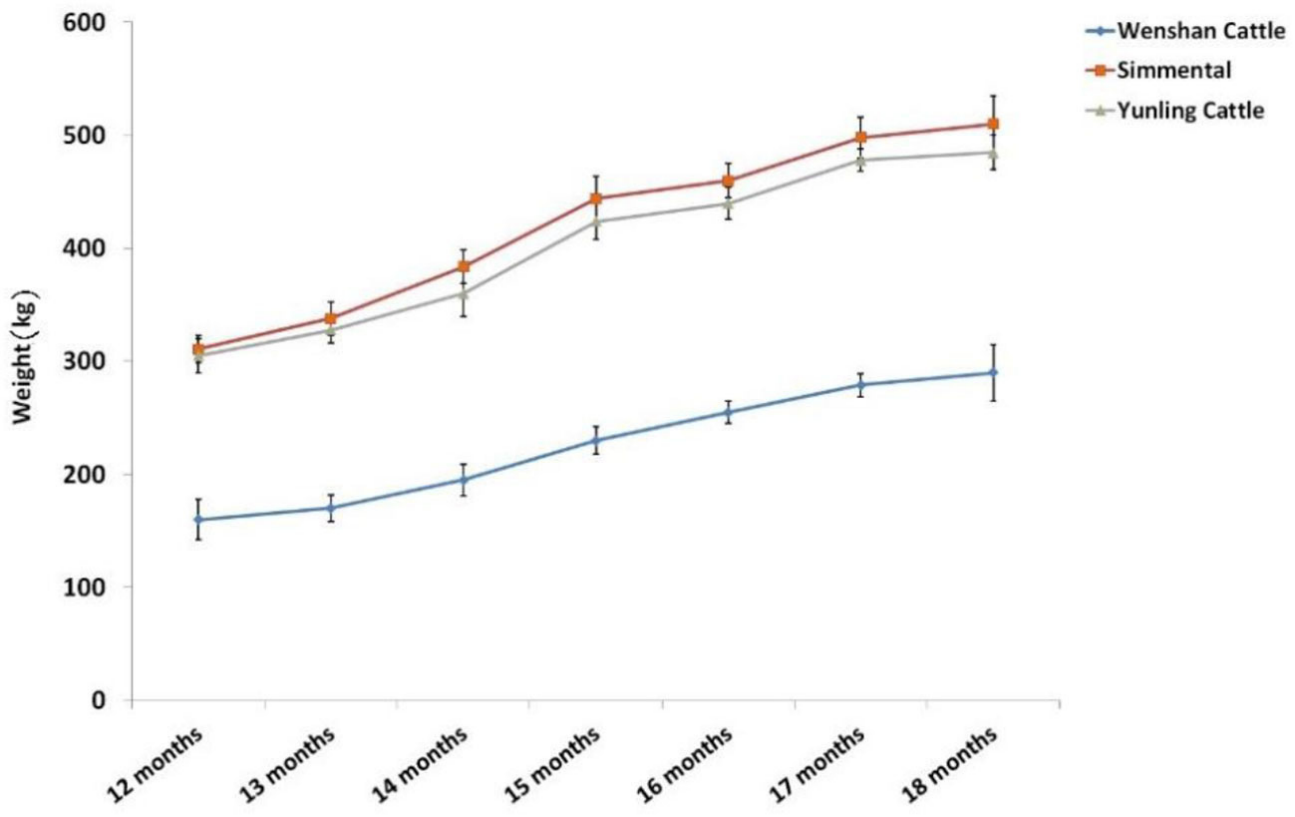
Trend of body weight change at different growth periods in the three cattle breeds. Data were presented as means ± SD of at least three independent experiments.

Body weight of Simmental and Yunling cattle was significantly greater than that of Wenshan cattle (*P* < 0.05; Table 2). The growth rate between 1 and 15 months of age was faster than that observed after 17 months of age regardless of breed (Figure 1).

**Table 2:** Body mass index at different growth periods in three cattle breeds.

**Age (months)**	**Breed**		
	**Yunling cattle (kg)**	**Wenshan cattle (kg)**	**Simmental cattle (kg)**
12	305.2 ± 19.1^b^	160.3 ± 32.2^a^	312.2 ± 38.3^b^
13	328.1 ± 14.4^b^	176.6 ± 32.5^a^	345.7 ± 35.5^b^
14	352.8 ± 14.8^b^	200.6 ± 37.9^a^	381.2 ± 28.8^b^
15	425.0 ± 15.7^b^	253.5 ± 48.7^a^	453.4 ± 32.3^b^
16	444.4 ± 98.0^b^	273.2 ± 46.0^a^	469.4 ± 34.5^b^
17	478.6 ± 10.4^b^	300.8 ± 48.9^a^	505.4 ± 41.5^b^
18	487.8 ± 14.0^b^	312.7 ± 50.1^a^	526.2 ± 38.6^b^

*Means with different letters (a, b) differ (P < 0.05). Data were presented as means ± SD of at least three independent experiments*.

At the age of 12 and 18 months, body size indexes including body height, body slant length, chest width, chest depth, chest circumference, abdominal circumference, hip circumference, waist angle width, ischial end width, tube circumference, and rump length of Simmental and Yunling cattle were significantly higher than those of Wenshan cattle (*P* < 0.05); however, there were no significant differences between Simmental and Yunling cattle (*P* > 0.05, Supplementary Table 1). In addition, the growth rate of Wenshan cattle was significantly higher than that of Simmental and Yunling cattle at the age of 12 and 18 months (*P* < 0.05).

Differences were detected in hormones associated with growth and development among Wenshan, Simmental, and Yunling cattle. Yunling cattle had the highest concentrations of insulin (INS), TSH, T4, T3, GH, and IGF-1 in plasma at the age of 12 months (*P* < 0.05; Supplementary Table 1). Additionally, T4 and INS levels in Yunling cattle were significantly higher than those in Wenshan cattle and Simmental cattle at the age of 18 months (*P* < 0.05; Table 3). Yunling cattle and Simmental cattle had greater concentrations of TSH, T3, GH, and IGF-1 than Wenshan cattle (*P* < 0.05). The hormone levels in Wenshan cattle showed a decreasing tendency from 12 to 18 months of age. By contrast, they showed an opposite trend in Simmental cattle and Yunling cattle.

**Table 3:** Growth hormone levels of three cattle breeds at the age of 18 months.

**Hormones**	**Breed**		
	**Wenshan cattle**	**Simmental cattle**	**Yunling cattle**
TSH, μIU/L	339.7 ± 11.2^a^	407.6 ± 15.1^b^	470.5 ± 12.4^b^
T4, μg/L	207.6 ± 11.3^a^	202.1 ± 5.2^a^	287.1 ± 42.9^b^
T3, pmol/L	49.3 ± 3.8^a^	59.8 ± 1.9^b^	54.6 ± 5.1^b^
GH, μg/L	23.3 ± 1.1^a^	27.1 ± 0.6^b^	28.7 ± 3.8^b^
INS, mIU/L	31.4 ± 1.5^a^	30.7 ± 1.4^a^	40.8 ± 3.3^b^
IGF-1, μg/L	18.5 ± 0.1^a^	22.4 ± 0.9^b^	23.1 ± 3.8^b^

*Means with different letters (a, b) differ (P < 0.05). Data were presented as means ± SD of at least three independent experiments*.

**Figure 2:**
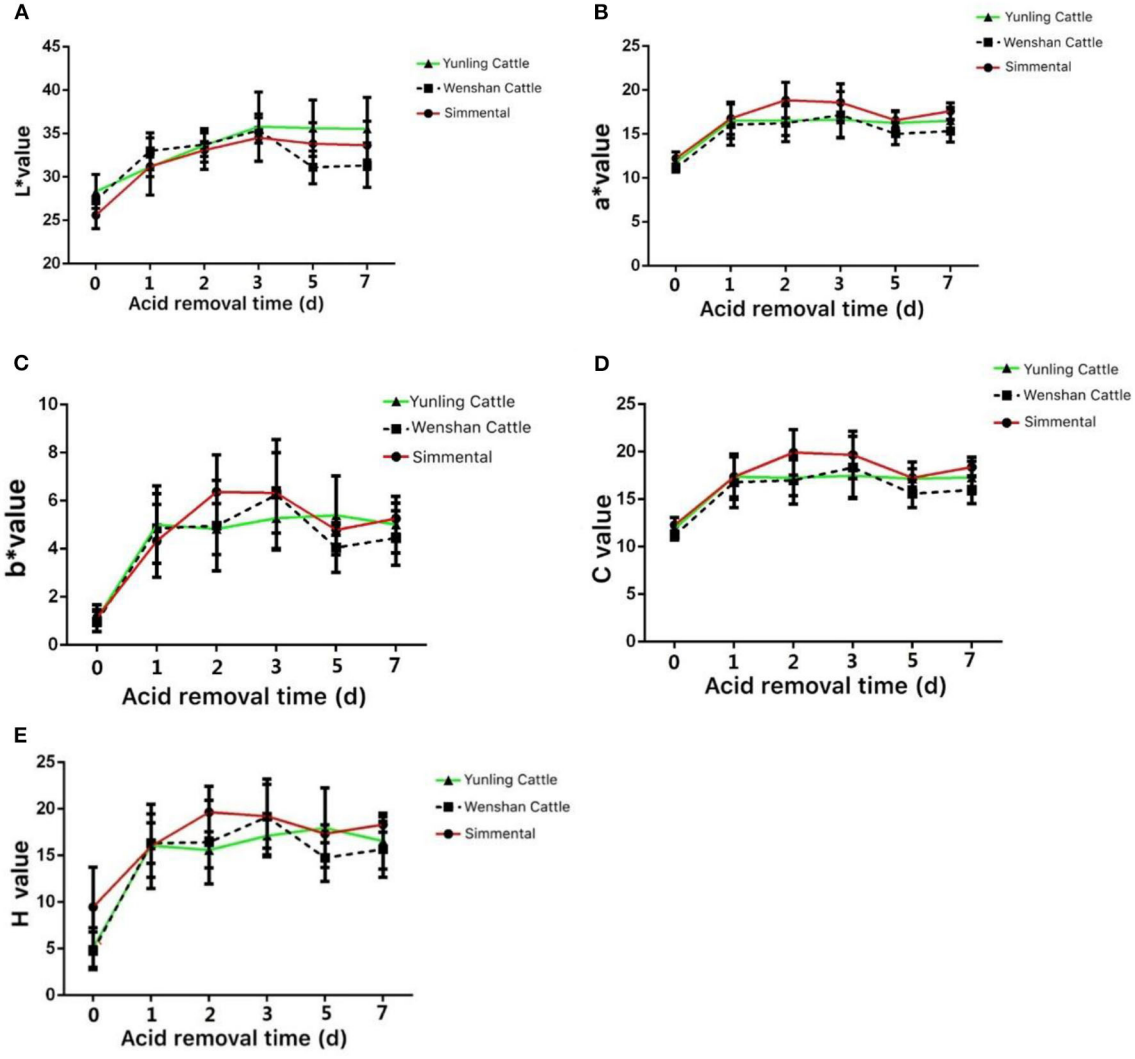
Meat color at different growth periods in the three cattle breeds. **(A)** Change in the L* value in the muscle aging process of the three cattle breeds; **(B)** change in the a* value during muscle aging in the three cattle breeds; **(C)** change in the b* value during muscle aging in the three cattle breeds; **(D)** change in the c* value during muscle aging in the three cattle breeds; **(E)** change in the *H* value during muscle aging in the three cattle breeds. Data were presented as means ± SD of at least three independent experiments.

The L^*^ values of Yunling cattle, Simmental cattle, and Wenshan cattle showed an increasing trend from 0 to 5 days postmortem. After 5 days postmortem, the L^*^ value of Wenshan cattle showed a significant reduction, and it displayed a slight decline in Yunling cattle and Simmental cattle (Figure 2A). The a^*^ value of Yunling cattle, Simmental cattle, and Wenshan cattle showed an increasing trend from 0 to 1 day after slaughter. After 5 days postmortem, Yunling cattle showed a steady state, while Simmental cattle and Wenshan cattle were characterized by a fluctuant trend. Overall, the a^*^ value of Simmental cattle was higher than that of the other two breeds postmortem (Figure 2B). The b^*^ value showed an increasing trend from 0 to 1 day in all breeds after slaughter. Moreover, the b^*^ value in Simmental cattle maintained an increasing trend 1–2 days after slaughter; conversely, it began to decline in the other two breeds (Figure 2C). It can be seen from Figure 2D that the *C* value of these three cattle breeds is highly similar to the a^*^ value. Additionally, it can be observed from Figure 2E that the *H* value of these cattle breeds is very similar to the b^*^ value.

**Figure 3:**
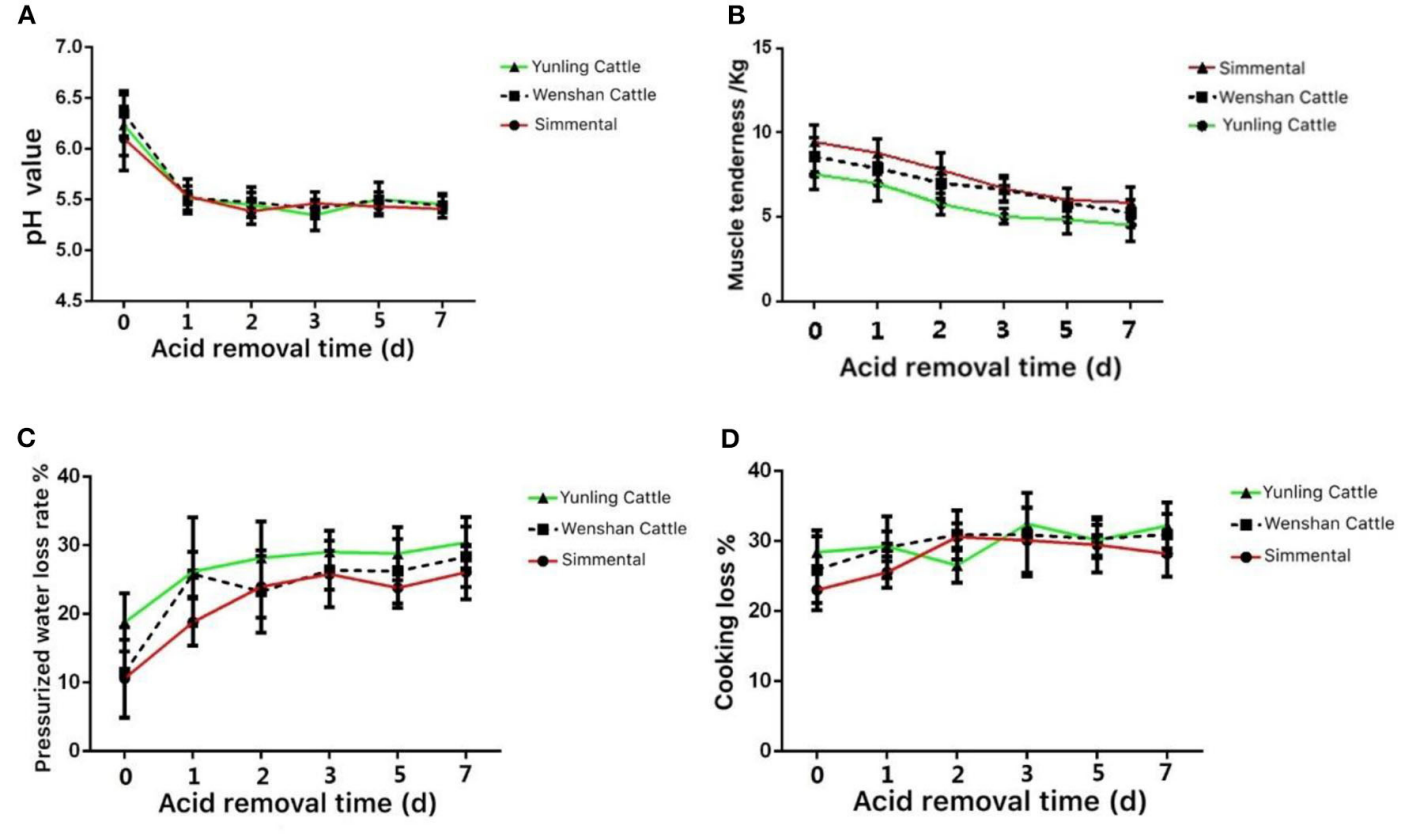
Meat quality of the three cattle breeds. **(A)** Change in pH value in the muscle aging process of different breeds of cattle; **(B)** change in muscle tenderness in the muscle aging process of different breeds of cattle; **(C)** change in pressurized water loss rate in the muscle aging process of different breeds of cattle; **(D)** change in weight while cooking in the muscle aging process of different breeds of cattle. Data were presented as means ± SD of at least three independent experiments.

The pH value in the muscle of Yunling cattle, Simmental cattle, and Wenshan cattle decreased significantly from 0 to 1 day postmortem. After 3 days postmortem, the change in pH tended to be stable (Figure 3A).

The shear force of Yunling cattle, Simmental cattle, and Wenshan cattle was all decreased between 0 and 5 days postmortem. Shear force decreased slightly during the first 5 days postmortem. As a whole, the shear force value at the same time point during acid excretion was Simmental cattle > Wenshan cattle > Yunling cattle. The results showed that the tenderness of Yunling cattle was better than that of Simmental cattle (Figure 3B).

The water loss rate of Yunling cattle, Simmental cattle, and Wenshan cattle increased markedly from 0 to 1 day postmortem. Overall, the water loss rate of Yunling cattle was higher than that of Simmental and Wenshan cattle during acid discharge (Figure 3C).

The cooking loss of Yunling cattle and Wenshan cattle increased, then it decreased followed by an increase. By contrast, cooking loss in Simmental cattle exhibited a clear increasing trend from 0 to 2 days and then began to decline (Figure 3D).

Correlations between the time of acid excretion and meat quality indexes (pH, water loss %, cooking loss %, muscle tenderness, L^*^ value, a^*^ value, and b^*^ value) in the three cattle breeds (Yunling cattle, Simmental cattle, and Wenshan cattle) were analyzed. The pH value and tenderness of Yunling cattle muscle were negatively correlated with the time of acid excretion (*P* < 0.01), whereas water loss rate, cooking loss, and meat color (L^*^ value, a^*^ value, and b^*^ value) were positively correlated with the time of acid excretion (all *P* < 0.05; Table 4). pH value had a significantly negative correlation with water loss rate and meat color (L^*^ value, a^*^ value, and b^*^ value) in Yunling cattle (*P* < 0.01), indicating that when the pH value of Yunling cattle muscle decreased, the water loss rate increased, and the meat color changed (Table 4).

**Table 4:** Correlation analysis of meat quality index in Yunling beef in the process of maturity.

**Index**	**Acid excretion time**	**pH**	**Water loss rate %**	**Cooking loss %**	**Tenderness**	L* value	a* value	b* value
Acid excretion time	1	−0.494**	0.399*	0.419*	−0.510**	0.607**	0.411*	0.472**
pH		1	−0.600**	−0.354	0.163	−0.622**	−0.677**	−0.662**
Water loss %			1	0.211	−0.264	0.539**	0.587**	0.514**
Cooking loss %				1	−0.084	0.316	0.135	0.221
Tenderness					1	−0.502**	−0.057	−0.067
L* value						1	0.591**	0.628**
a* value							1	0.932**
b* value								1

*Asterisk indicates significant difference (P < 0.05). Data were presented as means ± SD of at least three independent experiments*.

There was a significantly negative correlation between pH value and acid excretion time (*P* < 0.01), while there was no significantly negative correlation between pH value and muscle tenderness (*P* < 0.05; Table 5). The results showed that pH value decreased, but muscle tenderness did not change notably with prolonged acid excretion time. The pH value of Simmental cattle had a significantly negative correlation with water loss rate and meat color (L^*^ value, a^*^ value, and b^*^ value, Table 5).

**Table 5:** Correlation analysis of meat quality index in Simental beef in the process of maturity.

**Index**	**Acid excretion time**	**pH**	**Water loss rate %**	**Cooking loss %**	**Tenderness**	L* value	a* value	b* value
Acid excretion time	1	−0.546**	0.615**	0.367*	−0.410	0.553**	0.391*	0.417*
pH		1	−0.621**	−0.527**	−0.051	−0.728**	−0.629**	−0.653**
Water loss %			1	0.656**	−0.178	0.741**	0.769**	0.791**
Cooking loss %				1	0.181	0.590**	0.473**	0.558**
Meat tenderness					1	−0.214	−0.134	−0.109
L* value						1	0.712**	0.782**
a* value							1	0.962**
b* value								1

*Asterisk indicates significant difference (P < 0.05). Data were presented as means ± SD of at least three independent experiments*.

Acid excretion time was significantly negatively correlated with pH value and muscle tenderness (*P* < 0.01). Meanwhile, there was a significantly positive correlation between water loss (%) and acid excretion time (*P* < 0.01) and a significantly positive correlation between meat color (L^*^ value, a^*^ value, and b^*^ value) and water loss (%) (*P* < 0.05; Table 6). Thus, data suggest that with different time points, both pH value and muscle tenderness decrease, and the water loss rate under pressure increases; however, meat color and cooking loss may not be affected by acid discharge. The pH value of Wenshan beef showed a significantly negative correlation with water loss, cooking loss, and meat color (L^*^ value, a^*^ value, and b^*^ value, Table 6).

**Table 6:** Correlation analysis of meat quality index in Wenshan beef in the process of maturity.

**Index**	**Acid excretion time**	**pH**	**Water loss rate %**	**Cooking loss %**	**Tenderness**	L* value	a* value	b* value
Acid excretion time	1	−0.560**	0.534**	0.295	−0.598**	0.078	0.268	0.277
pH		1	−0.770**	−0.418*	0.336	−0.379*	−0.685**	−0.677**
Water loss %			1	0.353	−0.221	0.148	0.553**	0.536**
Cooking loss %				1	−0.121	0.104	0.187	0.169
Meat tenderness					1	−0.12	−0.256	−0.219
L* value						1	0.351	0.427*
a* value							1	0.966**
b* value								1

*Asterisk indicates significant difference (P < 0.05). Data were presented as means ± SD of at least three independent experiments*.

### Sample RNA Quality

Total RNA integrity and purity of six muscle samples (S1–S3 and y1–y3) satisfied sequencing standards (RIN ≥ 8.0; OD260 nm/OD280 nm ≥ 2.0; 28S/18S ≥ 1.6), which could be used for further RNA-Seq transcriptomic sequencing.

### Read Data Quality Control (Reads QC)

**Figure 4:**
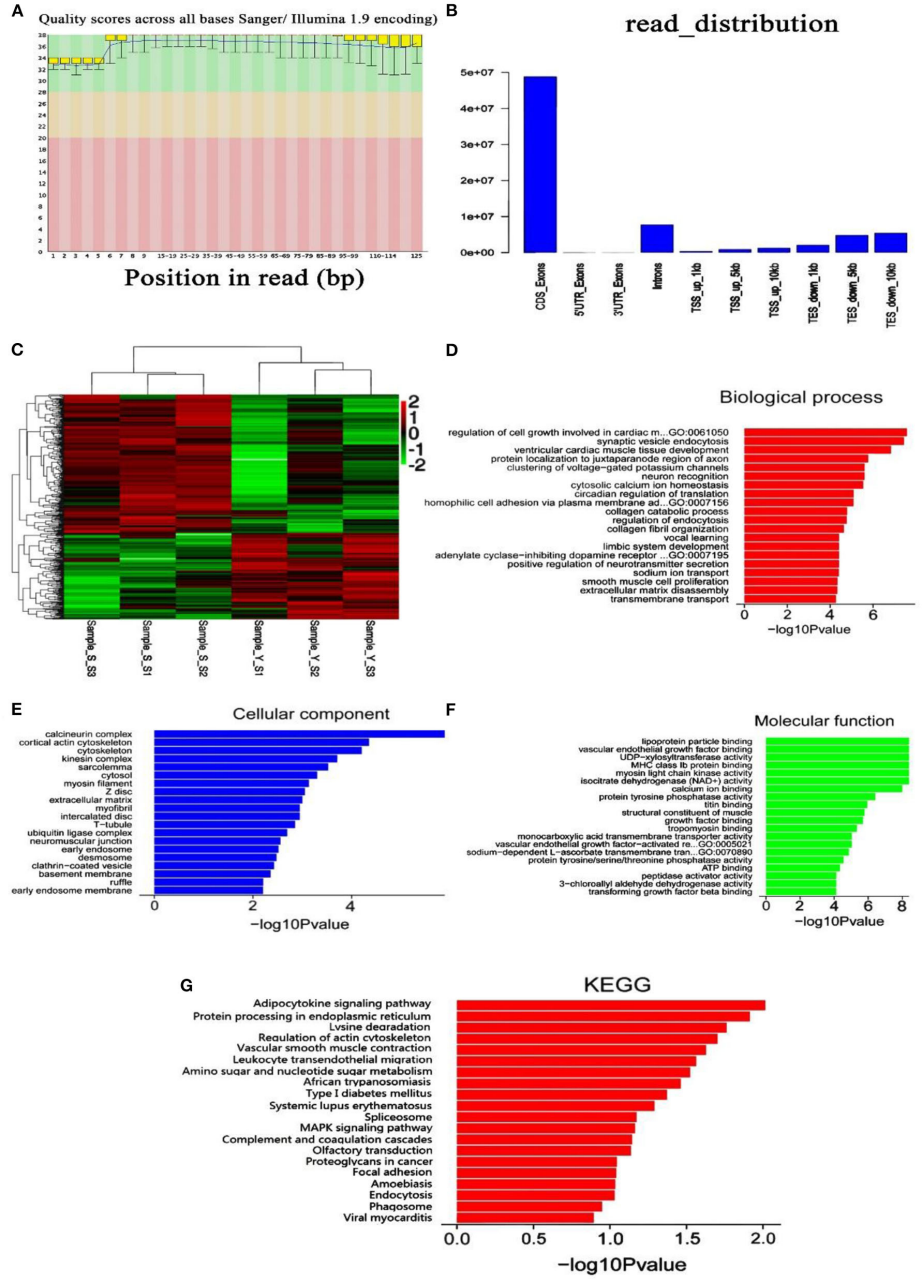
Transcriptome sequencing analysis. **(A)** Quality distribution of the original database group; **(B)** distribution of the original samples in different patterns; **(C)** cluster analysis of differentially expressed genes; **(D)** annotation of biological process; **(E)** annotation of cellular components; **(F)** annotation of molecular function; **(G)** annotation of KEGG pathways.

The base effective ratio and Q30 are above 90% (Supplementary Table 1). It can be seen from the base mass distribution diagram (Figure 4A) that the quality of sequencing data was above the average value. In addition to the first several bases, the transcripts obtained from the sequencing results had considerable fluctuation. The results showed that the sequencing data were suitable for further analysis.

### Sequencing Quality Control

In genome alignment, the reads across introns are divided into multiple tags. The number of tags was enriched in different gene elements to determine whether most of the tested samples fell into the coding sequence (CDS) region. It is shown in Figure 4B that most reads were matched to the CDS exon, which is in line with the results predicted by transcriptomic sequencing.

### Differentially Expressed Genes and Cluster Analysis

After sequencing, 890 differentially expressed genes were screened between the two groups according to the principle of *P* < 0.05 and log_2_ fold change >2. There were 356 upregulated genes and 534 downregulated genes (Figure 4C, Supplementary Material 1).

### GO Function and Enrichment Analysis of KEGG Pathways

Blast2GO software was used to annotate the GO function of differentially expressed genes and analyze the biological process, cellular component, and molecular function. The biological process module (Figure 4D) mainly includes the regulation of cell growth involved in cardiac muscle, the cell component module (Figure 4E) mainly includes the calcineurin complex, and the molecular function module (Figure 4F) mainly includes lipoprotein particle binding. The KEGG database was used to analyze differentially expressed genes involved in pathway enrichment (Figure 4G). The genes were enriched in 20 pathways, mainly in the adipocytokine signaling pathway and protein processing in the endoplasmic reticulum. Five significantly differentially expressed genes, namely *LEPR, STAT3, leptin, HSPA12A*, and *CAPN1*, were screened out.

### Quantitative PCR Verification of Differentially Expressed Genes

**Figure 5:**
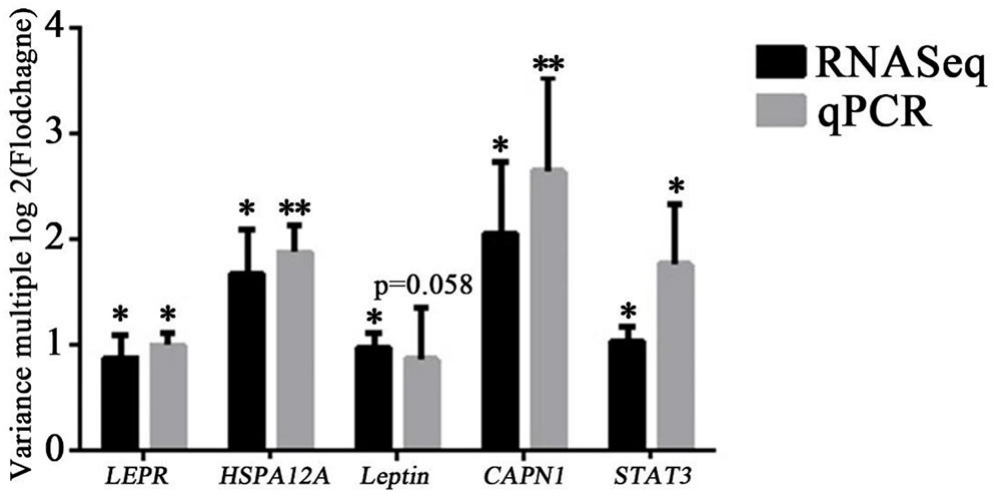
Verification of differentially expressed genes by qPCR. Data were presented as means ± SD of at least three independent experiments.

**Figure 6:**
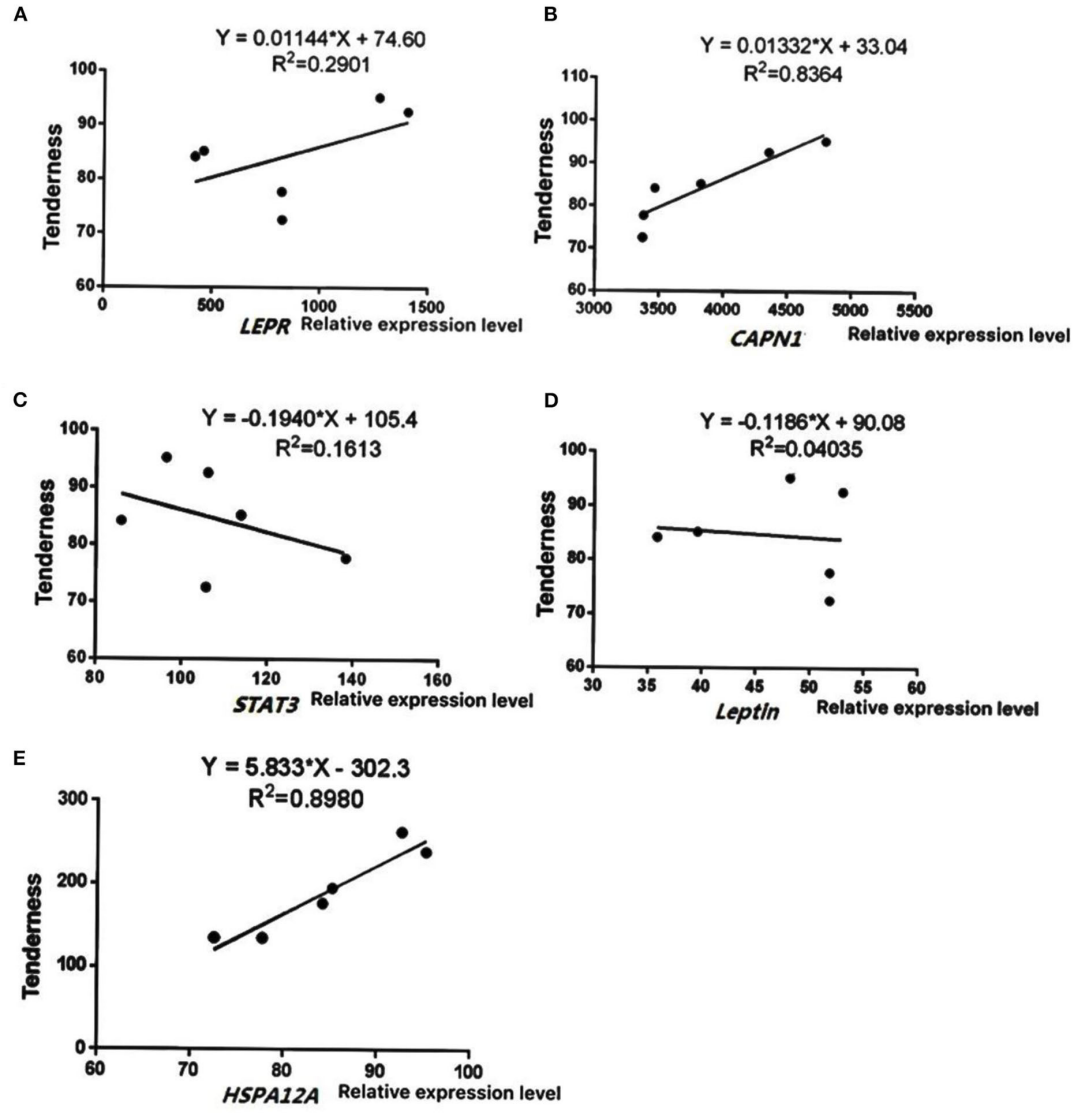
Correlation analysis between gene expression levels and tenderness. Data were presented as means ± SD of at least three independent experiments. **(A)** The correlation analysis between *LEPR* levels and muscle shear. **(B)** The correlation analysis between *CAPN1* levels and muscle shear. **(C)** The correlation analysis between *STAT3* levels and muscle shear. **(D)** The correlation analysis between *Leptin* levels and muscle shear. **(E)** The correlation analysis between *HSPA12A* levels and muscle shear.

To further verify the reliability of sequencing results, five key differentially expressed genes (*LEPR, STAT3, leptin, HSPA12A*, and *CAPN1*) were selected based on function and pathway enrichment in the present study. Results showed that the expression levels of the five genes in Simmental and Yunling cattle longissimus dorsi muscle were largely consistent with the transcriptomic RNA-Seq results, indicating that the sequencing results were reliable (Figure 5). The correlation analysis between expression levels and muscle shear force showed that *HSPA12A* and *CAPN1* were positively correlated with tenderness (*P* < 0.05), and the correlation coefficients were 0.8980 and 0.8364, respectively (Figure 6). Therefore, *HSPA12A* and *CAPN1* can be used as important candidate genes to further study how they regulate beef quality in Chinese native cattle in future research.

## Discussion

The food quality of beef is affected by many factors, including palatability, flavor, juiciness, and tenderness. Chinese native cattle resources are abundant (22, 23). These cattle have a unique origin system and excellent genetic resources and ecological adaptability (24, 25). These native cattle breeds have many specific genes and potentials for meat production (26, 27). The meat quality analysis of Chinese native cattle (Yunling cattle, Wenshan cattle), a new hybrid beef cattle bred by Chinese researchers, is still a research gap meat quality. To explore Chinese native cattle initially, indicators were measured associated with meat quality of Yunling, Simmental, and Wenshan cattle. Simmental is an important reference. This study measured a set of indicators of Yunling beef, Wenshan cattle, and other breed as a control (Simmental cattle, an excellent beef cattle breed in the world), including pH, muscle tenderness, water loss rate, cooking loss, correlation analysis of acid excretion time and meat quality indexes, and mRNA expression. Physical and chemical indexes, such as meat color, pH value, water loss rate, tenderness, and cooking loss, were measured. This study reveals the changing pattern of meat quality in Chinese native cattle during acid discharge and lays a theoretical foundation for further development of high-quality native beef in China. Meat color mainly reflects changes in biochemical, physiological, and microbiological properties in muscle (28). The observation of meat color is widely used as an effective way to evaluate meat quality by consumers. The L^*^ value of Yunling cattle was significantly higher than that of Simmental and Wenshan cattle after 2 days postmortem. By contrast, the a^*^ value in the muscle of Simmental cattle was higher than that of Yunling cattle and Wenshan cattle. The b^*^ value of Yunling cattle, Simmental cattle, and Wenshan cattle showed an increasing trend between 0 and 1 day postmortem. The b^*^ value of Simmental cattle continued to rise between 1 and 2 days postmortem, while it began to decrease in the other two cattle breeds. The increase of L^*^ value and the decrease of a^*^ value are mainly due to the infiltration of water in muscle, thereby increasing the reflection ability of light.

Cooking loss is an effective index to evaluate the water-holding capacity in muscle (29). It has been well-established that water is the major lost substance during cooking (30, 31). Generally, acid excretion time is positively related to cooking loss, which is partly explained by the changes of material composition, pH value, and heating temperature in muscle. The cooking loss of Yunling cattle and Wenshan cattle fluctuated postmortem. For instance, Simmental cattle showed an obvious increasing trend during the first 2 days postmortem and then began to decline. Overall, the cooking loss of Yunling cattle was greater than that of Wenshan cattle and Simmental cattle. In addition, the shear force value of Simmental and Wenshan cattle showed a significant downward trend. After 5 days postmortem, the shear force values decreased slightly and tended to be stable. As a whole, the shear force value at the same time point is as follows: Simmental cattle > Wenshan cattle > Yunling cattle. Therefore, data suggest that the muscle tenderness of Yunling cattle was better than that of Simmental cattle. Various biochemical reactions still exist in muscle postmortem but mainly anaerobic metabolism, constantly generating plenty of lactic acids and other substances, resulting in decreased pH value in muscle (32, 33). It was found that the tenderness of muscle was the highest at the beginning of slaughter and then reduced along with the decrease of pH. In the current study, the pH value of Yunling cattle, Simmental cattle, and Wenshan cattle decreased in the course of 0–7 days postmortem. In addition, changes in meat color, water-holding capacity, microbial growth rate, and protein solubility are all affected by decreased pH.

With the development of high-throughput sequencing technology, transcriptomic RNA-Seq sequencing has been widely used to determine gene expression levels in different species or tissues (34, 35). Bioinformatics is applied to elucidate the molecular mechanism on the formation of specific traits, which provides theoretical guidance for cattle breeding in the future. With the improvement of living standards, consumers are increasingly pursuing high-quality beef. To meet consumers' needs, it is highly important to cultivate beef breeds with good meat quality. Different growth traits, meat quality, and flavor in the three cattle breeds suggest potentially different mechanisms on regulating meat quality among different breeds. Therefore, we further analyzed differentially expressed genes in the longissimus dorsi muscle between Chinese native cattle and Simmental cattle. It is noteworthy that we found two important regulatory pathways (e.g., adipocytokine signaling pathway and protein processing in the endoplasmic reticulum) and screened some important differentially expressed genes, such as *LEPR, STAT3, leptin, HSPA12A*, and *CAPN1*. LEPR is a key receptor for leptin (36). STAT3, a signal transducer and activator of transcription, plays an important role in modulating cell proliferation, differentiation, and migration (37). *CAPN1*, as a cysteine-sparse endopeptidase, regulates biological processes of muscle growth and development, cell differentiation and apoptosis, and signal transduction (38). It was reported that calpain was the main enzyme that degraded muscle fiber, and enzyme activity was closely related to the change of meat tenderness. Moreover, it was also found that calpain could effectively improve feed efficiency in cattle. Heat shock proteins are proteins produced by stimulation of the external environment (39). Generally, different proteins in this family are highly conserved. These proteins are important nonspecific cytoprotective proteins that have a crucial influence on the maintenance of cell metabolism, apoptosis, and the stability of the internal environment. Thus, based on the correlation analysis between gene expression levels and tenderness, *HSPA12A* and *CAPN1* genes may play a critical role in changing native beef quality in China. Future research should be done using RNA interference and gene knockout technology at the cellular level to further clarify the specific functions of these target genes.

## Conclusion

Overall, potential genetic markers related to meat quality in Chinese native cattle were selected, which can provide a theoretical reference for genetic improvement and breeding in cattle in the future.

## Data Availability Statement

The original contributions presented in the study are publicly available. This data can be found at https://www.ncbi.nlm.nih.gov/ using the following codes: SRR13156118, SRR13156117, SRR13156116, SRR13156154, SRR13156153, SRR13156152, SRR13156151, SRR13156150, SRR13156149.

## Ethics Statement

The animal study was reviewed and approved by Institutional Animal Care and Use Committee of the College of Animal Science and Technology, Yangzhou University, Yang Zhou, China.

## Author Contributions

XM, ZG, and ZY: conceptualization and writing—review and editing. YL and CZ: methodology. ZC, YM, and CZ: investigation. ZY: resources, project administration, and funding acquisition. XM: writing—original draft preparation. ZC: supervision. All authors contributed to the article and approved the submitted version.

## Conflict of Interest

The authors declare that the research was conducted in the absence of any commercial or financial relationships that could be construed as a potential conflict of interest.
