# Supplementary material for: Longissimus Dorsi Muscle Transcriptomic Analysis of Simmental and Chinese Native Cattle Differing in Meat Quality
Source: Front Vet Sci. 2020 Dec 15;7:601064. doi: 10.3389/fvets.2020.601064 (PMC7770222; doi:10.3389/fvets.2020.601064)
Supplement: Supplementary file 1 [file Table_1.pdf]

TABLE 1

Sample name	Gene name	logFC	PValue
Simmental VS Wenshan	ENSBTAG00000046849	-2.774002614	1.90E-06
Simmental VS Wenshan	ENSBTAG00000018542	-2.743076525	0.000459
Simmental VS Wenshan	ADIPOQ	-2.221084144	0.005432
Simmental VS Wenshan	ENSBTAG00000012533	-2.21739156	0.008008
Simmental VS Wenshan	ENSBTAG00000047577	-2.136035716	0.021817
Simmental VS Wenshan	ENSBTAG00000030909	-2.065208405	0.016046
Simmental VS Wenshan	RPP14	-2.028555504	0.034344
Simmental VS Wenshan	CDCA3	-1.758282259	0.007364
Simmental VS Wenshan	C1orf146	-1.7216973	0.001544
Simmental VS Wenshan	ENSBTAG00000026543	-1.71113837	0.015403
Simmental VS Wenshan	S1PR1	-1.677724536	0.029072
Simmental VS Wenshan	GLMP	-1.675315277	0.001503
Simmental VS Wenshan	EBP	-1.629675637	0.024765
Simmental VS Wenshan	DND1	-1.5929888	0.00958
Simmental VS Wenshan	ENSBTAG00000025441	-1.584361238	0.002129
Simmental VS Wenshan	EGR4	-1.576890348	0.006567
Simmental VS Wenshan	ENSBTAG00000038227	-1.567466406	0.01232
Simmental VS Wenshan	SNRNP35	-1.560524207	0.02365
Simmental VS Wenshan	PCDHA5	-1.55559847	0.005881
Simmental VS Wenshan	ANXA7	-1.54969325	0.040854
Simmental VS Wenshan	ENSBTAG00000037558	-1.540975147	0.016327
Simmental VS Wenshan	ENSBTAG00000012822	-1.539850059	0.018422
Simmental VS Wenshan	C12orf29	-1.522650629	0.038335
Simmental VS Wenshan	NDNF	-1.518587577	0.032944
Simmental VS Wenshan	PGAM2	-1.50097682	0.021682
Simmental VS Wenshan	LRP2BP	-1.486575197	0.006753
Simmental VS Wenshan	MED18	-1.479973284	0.028674
Simmental VS Wenshan	METTL12	-1.474441001	0.044959
Simmental VS Wenshan	PSPN	-1.453532346	0.020694
Simmental VS Wenshan	CAV3	-1.453186783	9.29E-07
Simmental VS Wenshan	ENSBTAG00000023697	-1.444885663	0.024268
Simmental VS Wenshan	ENSBTAG00000045990	-1.444852917	0.016976
Simmental VS Wenshan	NMUR1	-1.443108601	1.33E-05
Simmental VS Wenshan	ENSBTAG00000046456	-1.441672724	0.00365
Simmental VS Wenshan	MTERF4	-1.438817282	0.016737
Simmental VS Wenshan	ENSBTAG00000001150	-1.433777471	0.006869
Simmental VS Wenshan	ENSBTAG00000007354	-1.431242942	0.035678
Simmental VS Wenshan	ENSBTAG00000024849	-1.425031328	0.000982
Simmental VS Wenshan	RNF26	-1.403115558	0.005878
Simmental VS Wenshan	ENSBTAG00000047258	-1.39938956	0.043959

Simmental VS Wenshan	CLDN1	-1.397644236	0.042227
Simmental VS Wenshan	ENSBTAG00000034611	-1.396146708	0.043559
Simmental VS Wenshan	SPRYD4	-1.392285003	0.042707
Simmental VS Wenshan	CD68	-1.389481012	0.022595
Simmental VS Wenshan	CDK13	-1.383167471	2.78E-07
Simmental VS Wenshan	TPPP2	-1.380209375	0.019604
Simmental VS Wenshan	ENSBTAG00000012169	-1.370417168	0.019076
Simmental VS Wenshan	RPS5	-1.336118782	0.014378
Simmental VS Wenshan	TUBA1B	-1.314946131	0.045767
Simmental VS Wenshan	RGS1	-1.296795185	0.022818
Simmental VS Wenshan	ENSBTAG00000001394	-1.286009585	0.025721
Simmental VS Wenshan	S100A4	-1.268153949	0.04084
Simmental VS Wenshan	ENSBTAG00000045867	-1.250334183	0.001568
Simmental VS Wenshan	CLEC2L	-1.245935729	7.50E-05
Simmental VS Wenshan	ARL4D	-1.24304303	0.007941
Simmental VS Wenshan	TUBE1	-1.241614905	0.031205
Simmental VS Wenshan	GRM6	-1.239368169	0.000818
Simmental VS Wenshan	ENSBTAG00000048226	-1.223611977	0.029223
Simmental VS Wenshan	CD207	-1.220254803	0.016091
Simmental VS Wenshan	BHMT2	-1.21462785	0.017253
Simmental VS Wenshan	COQ10A	-1.210841081	0.022108
Simmental VS Wenshan	ENSBTAG00000032702	-1.197070256	0.01283
Simmental VS Wenshan	ENSBTAG00000036082	-1.1880552	0.017081
Simmental VS Wenshan	LGALS3	-1.187536964	0.001153
Simmental VS Wenshan	KCTD14	-1.186527793	0.040551
Simmental VS Wenshan	GORAB	-1.185474242	0.032289
Simmental VS Wenshan	LYG2	-1.178258836	0.01589
Simmental VS Wenshan	ENSBTAG00000047304	-1.173166017	0.046518
Simmental VS Wenshan	PTRH2	-1.158140979	0.010022
Simmental VS Wenshan	ENSBTAG00000047997	-1.149998512	0.049251
Simmental VS Wenshan	LZTS1	-1.147314522	0.002571
Simmental VS Wenshan	IFI44	-1.143178514	0.016294
Simmental VS Wenshan	C2CD4C	-1.141408237	0.042101
Simmental VS Wenshan	C1orf110	-1.140993252	0.017658
Simmental VS Wenshan	SLC16A1	-1.137351574	0.006653
Simmental VS Wenshan	BAZ1A	-1.13507668	0.005764
Simmental VS Wenshan	GPR132	-1.113875238	0.025338
Simmental VS Wenshan	ZNF3	-1.101705644	0.003203
Simmental VS Wenshan	ENSBTAG00000019273	-1.100831842	0.009302
Simmental VS Wenshan	UBN2	-1.095044392	0.000104
Simmental VS Wenshan	TNFSF14	-1.085749318	0.018061
Simmental VS Wenshan	CHRM5	-1.072955845	0.015752
Simmental VS Wenshan	ENSBTAG00000006621	-1.06788132	0.031103

Simmental VS Wenshan	ANKRD60	-1.062715844	0.001192
Simmental VS Wenshan	PARP2	-1.062485464	0.047241
Simmental VS Wenshan	CREB3L4	-1.062164325	0.016136
Simmental VS Wenshan	ENSBTAG00000011985	-1.057629763	0.000125
Simmental VS Wenshan	WRN	-1.056720582	0.000118
Simmental VS Wenshan	NMRAL1	-1.055716591	0.047389
Simmental VS Wenshan	C10orf10	-1.054822135	0.039767
Simmental VS Wenshan	MIER3	-1.054323417	0.02777
Simmental VS Wenshan	CCDC106	-1.051184492	0.014701
Simmental VS Wenshan	RAB12	-1.04886729	0.017829
Simmental VS Wenshan	TFF1	-1.040222578	0.010416
Simmental VS Wenshan	BPIFA1	-1.034570672	0.030063
Simmental VS Wenshan	NR1I3	-1.019643021	0.016982
Simmental VS Wenshan	GPRIN2	-1.018095338	0.008641
Simmental VS Wenshan	ACKR2	-1.009666529	0.019413
Simmental VS Wenshan	MT4	-1.009073343	0.040855
Simmental VS Wenshan	PTI	-1.002533751	0.010411
Simmental VS Wenshan	ENSBTAG00000006354	-1.000734038	0.001048
Simmental VS Wenshan	C1orf216	-0.997276732	0.026471
Simmental VS Wenshan	ZBTB14	-0.996280703	0.014703
Simmental VS Wenshan	FAM96B	-0.989521362	0.028246
Simmental VS Wenshan	MID2	-0.984095947	0.049806
Simmental VS Wenshan	TFAP2C	-0.982714101	0.015481
Simmental VS Wenshan	ENSBTAG00000046325	-0.982363387	0.012379
Simmental VS Wenshan	PPM1M	-0.97590255	0.000959
Simmental VS Wenshan	ENSBTAG00000034940	-0.967067896	0.013288
Simmental VS Wenshan	BARX2	-0.96088698	0.005093
Simmental VS Wenshan	CD59	-0.947258341	0.005405
Simmental VS Wenshan	TIPIN	-0.94391561	0.024481
Simmental VS Wenshan	TCTN2	-0.941784594	0.000834
Simmental VS Wenshan	ANKRD54	-0.941345396	0.008993
Simmental VS Wenshan	GAS2L2	-0.932549228	0.001526
Simmental VS Wenshan	ENSBTAG00000034531	-0.916305755	0.043123
Simmental VS Wenshan	ENSBTAG00000004374	-0.915604177	0.023844
Simmental VS Wenshan	ENSBTAG00000047181	-0.913717328	6.85E-05
Simmental VS Wenshan	ENSBTAG00000012146	-0.910092478	0.037475
Simmental VS Wenshan	METAP2	-0.907470246	0.028197
Simmental VS Wenshan	TFF2	-0.906816193	0.030853
Simmental VS Wenshan	PRKAG3	-0.906534981	0.0145
Simmental VS Wenshan	ENSBTAG00000047668	-0.903788951	0.013149
Simmental VS Wenshan	ENSBTAG00000021111	-0.89999837	0.022139
Simmental VS Wenshan	LCN9	-0.898450668	0.026201
Simmental VS Wenshan	ENSBTAG00000024874	-0.897052272	0.007408

Simmental VS Wenshan	HTR6	-0.894273401	0.000718
Simmental VS Wenshan	APOC4	-0.89131609	0.032266
Simmental VS Wenshan	DLST	-0.89002669	0.021942
Simmental VS Wenshan	C8orf22	-0.876949041	0.031736
Simmental VS Wenshan	ENSBTAG00000040038	-0.875049062	0.013791
Simmental VS Wenshan	LEPR	-0.867907564	0.01853
Simmental VS Wenshan	RCCD1	-0.859730532	0.007613
Simmental VS Wenshan	RHOC	-0.855715577	0.00255
Simmental VS Wenshan	EMX2	-0.854634041	0.025227
Simmental VS Wenshan	HOMER3	-0.853922783	0.046659
Simmental VS Wenshan	ZNF444	-0.841747408	0.01002
Simmental VS Wenshan	CCDC166	-0.824697809	0.04406
Simmental VS Wenshan	VTCN1	-0.824556924	0.031209
Simmental VS Wenshan	FNDC5	-0.824442922	0.041625
Simmental VS Wenshan	BSCL2	-0.822778922	0.040831
Simmental VS Wenshan	ZDHHC22	-0.822626242	0.03197
Simmental VS Wenshan	ALKBH6	-0.821747644	0.023866
Simmental VS Wenshan	ENSBTAG00000019423	-0.821740196	0.009187
Simmental VS Wenshan	ENSBTAG00000012971	-0.818691442	0.049148
Simmental VS Wenshan	HDGF	-0.815725378	0.023536
Simmental VS Wenshan	SIAH2	-0.8152533	0.037308
Simmental VS Wenshan	SCRIB	-0.80383558	0.000163
Simmental VS Wenshan	LRRC73	-0.802208899	0.044518
Simmental VS Wenshan	LAMA4	-0.801416378	0.000153
Simmental VS Wenshan	ENSBTAG00000013720	-0.791576735	0.029686
Simmental VS Wenshan	DERL1	-0.789908254	0.040583
Simmental VS Wenshan	GOT1L1	-0.78742505	0.008315
Simmental VS Wenshan	BLCAP	-0.78266183	0.014204
Simmental VS Wenshan	PXMP4	-0.77921514	0.011179
Simmental VS Wenshan	ENSBTAG00000039691	-0.77360208	0.003807
Simmental VS Wenshan	ENSBTAG00000016748	-0.766601101	0.0117
Simmental VS Wenshan	SERPING1	-0.763304259	0.002272
Simmental VS Wenshan	TESK1	-0.761396621	0.023336
Simmental VS Wenshan	PLK1	-0.760373383	0.021054
Simmental VS Wenshan	GP5	-0.754870473	0.006702
Simmental VS Wenshan	CDC42EP1	-0.753847085	0.023781
Simmental VS Wenshan	NELFE	-0.753189142	0.014889
Simmental VS Wenshan	ENSBTAG00000020454	-0.751506173	0.03248
Simmental VS Wenshan	ENSBTAG00000023309	-0.748517675	0.024455
Simmental VS Wenshan	SASS6	-0.748323509	0.045414
Simmental VS Wenshan	CACNB3	-0.746644932	0.032076
Simmental VS Wenshan	GARNL3	-0.746107754	0.0031
Simmental VS Wenshan	CTNNB1	-0.742060982	0.013957

Simmental VS Wenshan	RNF103	-0.739230913	0.040096
Simmental VS Wenshan	ANKRD10	-0.736371463	0.043389
Simmental VS Wenshan	CTDSP1	-0.732071209	0.048319
Simmental VS Wenshan	RNF10	-0.730515047	0.049459
Simmental VS Wenshan	PRRC1	-0.727968291	0.035678
Simmental VS Wenshan	ENSBTAG00000046835	-0.727397124	0.035502
Simmental VS Wenshan	MZF1	-0.726237465	0.02973
Simmental VS Wenshan	ENSBTAG00000035129	-0.72116347	0.00347
Simmental VS Wenshan	C11orf84	-0.718496653	0.0359
Simmental VS Wenshan	FAM92B	-0.714585468	0.038306
Simmental VS Wenshan	DUSP7	-0.713168955	0.043993
Simmental VS Wenshan	LRRC31	-0.713045957	0.047649
Simmental VS Wenshan	MTIF3	-0.708874252	0.018773
Simmental VS Wenshan	FEM1A	-0.707793602	0.03127
Simmental VS Wenshan	TALDO1	-0.703468365	0.035857
Simmental VS Wenshan	NAT9	-0.701802224	0.035081
Simmental VS Wenshan	GLIS2	-0.700821031	0.028806
Simmental VS Wenshan	ENSBTAG00000047900	-0.70081202	0.00237
Simmental VS Wenshan	FAM111B	-0.698349374	0.021196
Simmental VS Wenshan	HSD17B3	-0.69474165	0.015466
Simmental VS Wenshan	FAM204A	-0.694402268	0.031136
Simmental VS Wenshan	CCDC176	-0.690816917	0.011448
Simmental VS Wenshan	DDX39A	-0.690229321	0.016508
Simmental VS Wenshan	KXD1	-0.68864518	0.023769
Simmental VS Wenshan	MIEF2	-0.683834569	0.048855
Simmental VS Wenshan	ACHE	-0.676029226	0.042895
Simmental VS Wenshan	CCR9	-0.671768486	0.00143
Simmental VS Wenshan	CYB5RL	-0.669078477	0.021207
Simmental VS Wenshan	PNCK	-0.667368667	0.045657
Simmental VS Wenshan	ENSBTAG00000045928	-0.665731949	0.019504
Simmental VS Wenshan	TPRA1	-0.657458174	0.009242
Simmental VS Wenshan	CTHRC1	-0.656728554	0.036725
Simmental VS Wenshan	ENSBTAG00000045964	-0.653250582	0.049279
Simmental VS Wenshan	AMH	-0.648317365	0.031224
Simmental VS Wenshan	ZHX2	-0.646221659	0.03993
Simmental VS Wenshan	RCAN1	-0.646022212	0.037917
Simmental VS Wenshan	NPTX1	-0.64046747	0.038542
Simmental VS Wenshan	SPATA2L	-0.637999746	0.029706
Simmental VS Wenshan	OPRK1	-0.634021605	0.046934
Simmental VS Wenshan	ITGB1BP1	-0.633253101	0.0231
Simmental VS Wenshan	CHFR	-0.625675784	0.041302
Simmental VS Wenshan	PLIN4	-0.624303161	0.020097
Simmental VS Wenshan	LRRC63	-0.62289917	0.031808

Simmental VS Wenshan	DISP2	-0.620836338	0.00747
Simmental VS Wenshan	LDLRAD1	-0.61749609	0.035623
Simmental VS Wenshan	ILDR1	-0.615272834	0.033758
Simmental VS Wenshan	DOHH	-0.615082799	0.029717
Simmental VS Wenshan	PRR22	-0.610898741	0.044365
Simmental VS Wenshan	TNFRSF4	-0.608790056	0.037926
Simmental VS Wenshan	C14orf79	-0.607606918	0.024693
Simmental VS Wenshan	ENSBTAG00000038497	-0.606069827	0.004386
Simmental VS Wenshan	PLEKHG4	-0.605745147	0.027173
Simmental VS Wenshan	CYP24A1	-0.602128964	0.013462
Simmental VS Wenshan	FTCDNL1	-0.590532105	0.040798
Simmental VS Wenshan	XRCC6BP1	-0.588905376	0.020971
Simmental VS Wenshan	SH3GL3	-0.588464705	0.022495
Simmental VS Wenshan	DDX54	-0.586131097	0.006741
Simmental VS Wenshan	SERPINB7	-0.585237806	0.046398
Simmental VS Wenshan	TNFRSF1A	-0.584034486	0.043351
Simmental VS Wenshan	MATN4	-0.583247315	0.019517
Simmental VS Wenshan	TP53BP1	-0.58291819	0.000897
Simmental VS Wenshan	PRSS21	-0.582276623	0.017261
Simmental VS Wenshan	ROPN1L	-0.577614322	0.04873
Simmental VS Wenshan	KDM6B	-0.576971185	0.003129
Simmental VS Wenshan	TRMT2A	-0.56453542	0.04431
Simmental VS Wenshan	GATA3	-0.553723764	0.007105
Simmental VS Wenshan	GTF3C1	-0.549810949	0.007539
Simmental VS Wenshan	PHYHIP	-0.540365197	0.034523
Simmental VS Wenshan	ENSBTAG00000006508	-0.53559061	0.001319
Simmental VS Wenshan	LFNG	-0.530332246	0.018686
Simmental VS Wenshan	DLK1	-0.528317143	0.04
Simmental VS Wenshan	VARS2	-0.522617847	0.028144
Simmental VS Wenshan	ETV4	-0.522596646	0.047919
Simmental VS Wenshan	C1orf177	-0.520817659	0.014112
Simmental VS Wenshan	POLD1	-0.517288757	0.006917
Simmental VS Wenshan	TAF4	-0.516709074	0.001918
Simmental VS Wenshan	EMILIN3	-0.516665853	0.043042
Simmental VS Wenshan	ZC3H12A	-0.515626494	0.011712
Simmental VS Wenshan	ATP1A2	-0.515606839	0.019061
Simmental VS Wenshan	ENSBTAG00000019790	-0.514860553	0.026459
Simmental VS Wenshan	ACP2	-0.513349805	0.038186
Simmental VS Wenshan	NELFCD	-0.512137964	0.038811
Simmental VS Wenshan	AGTRAP	-0.510989449	0.032657
Simmental VS Wenshan	PODN	-0.508052386	0.034976
Simmental VS Wenshan	RNPEP	-0.507929205	0.041743
Simmental VS Wenshan	ENSBTAG00000038030	-0.507151676	0.006965

Simmental VS Wenshan	COPG2	-0.500949525	0.018483
Simmental VS Wenshan	ENSBTAG00000020356	-0.500591371	0.01813
Simmental VS Wenshan	ENSBTAG00000013562	-0.500477475	0.020129
Simmental VS Wenshan	TMEM8C	-0.499681032	0.039833
Simmental VS Wenshan	PGF	-0.499449199	0.029634
Simmental VS Wenshan	ENSBTAG00000000573	-0.498918132	0.014392
Simmental VS Wenshan	TNFAIP8L1	-0.496147919	0.014314
Simmental VS Wenshan	ENSBTAG00000023079	-0.491237098	0.026849
Simmental VS Wenshan	ENSBTAG00000035333	-0.489888335	0.015262
Simmental VS Wenshan	CHRDL2	-0.488404439	0.038361
Simmental VS Wenshan	SDHA	-0.487015897	0.001126
Simmental VS Wenshan	ZYX	-0.486851904	0.0379
Simmental VS Wenshan	ITGA10	-0.484463457	0.035638
Simmental VS Wenshan	TMEM8A	-0.477439874	0.020441
Simmental VS Wenshan	AP5Z1	-0.476913994	0.00999
Simmental VS Wenshan	ENSBTAG00000040046	-0.471008681	0.046614
Simmental VS Wenshan	TRIP13	-0.46601573	0.049447
Simmental VS Wenshan	CADM3	-0.460186598	0.040432
Simmental VS Wenshan	POLR2E	-0.459511996	0.048627
Simmental VS Wenshan	DCAF6	-0.453967238	0.008678
Simmental VS Wenshan	RGS7	-0.453490357	0.0012
Simmental VS Wenshan	FAM184B	-0.451455935	0.042496
Simmental VS Wenshan	ZBTB21	-0.44951258	0.045582
Simmental VS Wenshan	ILF3	-0.447729455	0.013601
Simmental VS Wenshan	CAB39	-0.441498197	0.022703
Simmental VS Wenshan	ENSBTAG00000003408	-0.439865154	0.048129
Simmental VS Wenshan	ENSBTAG00000012981	-0.43962441	0.048796
Simmental VS Wenshan	BLM	-0.432542748	0.02338
Simmental VS Wenshan	JPH2	-0.432063208	0.028489
Simmental VS Wenshan	POLA2	-0.431484236	0.043165
Simmental VS Wenshan	CXorf57	-0.429471155	0.03277
Simmental VS Wenshan	NKD2	-0.426918781	0.034619
Simmental VS Wenshan	ENSBTAG00000003244	-0.426897928	0.043862
Simmental VS Wenshan	LGALS3BP	-0.425715349	0.024643
Simmental VS Wenshan	FUK	-0.425050905	0.021627
Simmental VS Wenshan	KCNQ4	-0.424706916	0.030724
Simmental VS Wenshan	VSX2	-0.421852293	0.020084
Simmental VS Wenshan	WDR82	-0.42071282	0.012825
Simmental VS Wenshan	SLC8B1	-0.407124006	0.022226
Simmental VS Wenshan	MRPL21	-0.404046871	0.047344
Simmental VS Wenshan	KIAA1919	-0.402808496	0.015085
Simmental VS Wenshan	TYK2	-0.401712421	0.023103
Simmental VS Wenshan	CABLES2	-0.398155328	0.040442

Simmental VS Wenshan	ARHGEF5	-0.397215426	0.032165
Simmental VS Wenshan	FERMT3	-0.392898832	0.031328
Simmental VS Wenshan	THBS2	-0.385752522	0.039464
Simmental VS Wenshan	ELAC2	-0.385050748	0.010157
Simmental VS Wenshan	ELAVL1	-0.38452354	0.015435
Simmental VS Wenshan	ENSBTAG00000008609	-0.383465684	0.036704
Simmental VS Wenshan	SYNM	-0.381242146	0.041963
Simmental VS Wenshan	ABCB10	-0.376240502	0.047517
Simmental VS Wenshan	ANPEP	-0.373105876	0.036607
Simmental VS Wenshan	SLC28A1	-0.367327388	0.012748
Simmental VS Wenshan	GPR157	-0.361444462	0.045885
Simmental VS Wenshan	MFSD4	-0.359631145	0.034734
Simmental VS Wenshan	KRBA1	-0.359278423	0.019025
Simmental VS Wenshan	ZNF541	-0.359222369	0.049082
Simmental VS Wenshan	WNT4	-0.349599541	0.039162
Simmental VS Wenshan	ENSBTAG00000034674	-0.34581532	0.045911
Simmental VS Wenshan	TCF3	-0.336932757	0.029101
Simmental VS Wenshan	GLB1	-0.334278742	0.048231
Simmental VS Wenshan	ABCB8	-0.331130751	0.048339
Simmental VS Wenshan	KCNH3	-0.326754545	0.028478
Simmental VS Wenshan	ST8SIA2	-0.31261661	0.040562
Simmental VS Wenshan	ZNF76	-0.30707904	0.044756
Simmental VS Wenshan	ZXDC	-0.28399392	0.049624
Simmental VS Wenshan	ROCK1	0.279998495	0.049544
Simmental VS Wenshan	CUBN	0.307100322	0.014184
Simmental VS Wenshan	ENSBTAG00000047121	0.309618119	0.040498
Simmental VS Wenshan	EPB41L3	0.315495832	0.046537
Simmental VS Wenshan	SYNE1	0.315508917	0.025575
Simmental VS Wenshan	ENSBTAG00000002007	0.319189968	0.046375
Simmental VS Wenshan	ATG12	0.328032631	0.023406
Simmental VS Wenshan	KIFC3	0.331779834	0.041304
Simmental VS Wenshan	ADGRV1	0.33281318	0.039009
Simmental VS Wenshan	SLC35F1	0.334355025	0.044913
Simmental VS Wenshan	ENSBTAG00000021064	0.335957632	0.048122
Simmental VS Wenshan	SLC25A29	0.339071726	0.046624
Simmental VS Wenshan	PIEZO2	0.339206817	0.040541
Simmental VS Wenshan	PLEKHG5	0.340629644	0.0347
Simmental VS Wenshan	IQGAP1	0.344385296	0.049878
Simmental VS Wenshan	PLCXD3	0.346218676	0.035034
Simmental VS Wenshan	OLA1	0.352006118	0.025636
Simmental VS Wenshan	AKAP7	0.352381808	0.046153
Simmental VS Wenshan	AVEN	0.352558051	0.022315
Simmental VS Wenshan	KCNH5	0.366828881	0.04662

Simmental VS Wenshan	WDR4	0.370091676	0.049806
Simmental VS Wenshan	APEH	0.372947483	0.040139
Simmental VS Wenshan	FNBP1	0.374623252	0.017221
Simmental VS Wenshan	DOCK1	0.382787959	0.004172
Simmental VS Wenshan	SPTLC3	0.38932177	0.025849
Simmental VS Wenshan	JADE1	0.390442033	0.044717
Simmental VS Wenshan	ENSBTAG00000047320	0.392909081	0.04514
Simmental VS Wenshan	ENSBTAG00000047595	0.405021941	0.048123
Simmental VS Wenshan	THBS4	0.405832455	0.033114
Simmental VS Wenshan	PARP12	0.409941753	0.037046
Simmental VS Wenshan	ENSBTAG00000017829	0.412583202	0.039647
Simmental VS Wenshan	RRAGD	0.414274327	0.030882
Simmental VS Wenshan	WDR60	0.414716064	0.001416
Simmental VS Wenshan	LRRC9	0.415333847	0.015672
Simmental VS Wenshan	SRGAP1	0.415422122	0.00578
Simmental VS Wenshan	ARHGEF26	0.415671146	0.02131
Simmental VS Wenshan	TMPRSS15	0.416010298	0.027104
Simmental VS Wenshan	ANKFN1	0.416019496	0.005271
Simmental VS Wenshan	OSBPL3	0.422265411	0.035111
Simmental VS Wenshan	SAMD5	0.422430766	0.014193
Simmental VS Wenshan	NTN4	0.426382138	0.022307
Simmental VS Wenshan	SLC39A8	0.42655339	0.007483
Simmental VS Wenshan	YBX1	0.427862589	0.022689
Simmental VS Wenshan	PLA2G4F	0.428884849	0.049547
Simmental VS Wenshan	DOCK9	0.430135644	0.004826
Simmental VS Wenshan	RTKN2	0.431772363	0.035287
Simmental VS Wenshan	ENSBTAG00000039470	0.432521091	0.004524
Simmental VS Wenshan	ENSBTAG00000005339	0.435553189	0.044641
Simmental VS Wenshan	LPO	0.444518552	0.016627
Simmental VS Wenshan	KIF1BP	0.44539927	0.047967
Simmental VS Wenshan	CALD1	0.445419872	0.034156
Simmental VS Wenshan	ENSBTAG00000020802	0.445432307	0.019034
Simmental VS Wenshan	BPI	0.445622271	0.049053
Simmental VS Wenshan	DNAH11	0.447687278	0.004465
Simmental VS Wenshan	GSAP	0.452099684	0.041081
Simmental VS Wenshan	ENSBTAG00000046276	0.45390905	0.008634
Simmental VS Wenshan	SLC44A5	0.458195855	0.001838
Simmental VS Wenshan	CCDC33	0.458970742	0.011444
Simmental VS Wenshan	GSTZ1	0.459553796	0.042605
Simmental VS Wenshan	ENSBTAG00000047638	0.459753252	0.017286
Simmental VS Wenshan	CEP126	0.461866221	0.028046
Simmental VS Wenshan	FMO4	0.462724999	0.046255
Simmental VS Wenshan	FAM104A	0.462854212	0.0498

Simmental VS Wenshan	C3orf70	0.463661717	0.037374
Simmental VS Wenshan	ATXN7L2	0.467306897	0.048384
Simmental VS Wenshan	UBE2H	0.467518467	0.015956
Simmental VS Wenshan	PTGFR	0.470909733	0.022015
Simmental VS Wenshan	ADAMTS3	0.471347571	0.0351
Simmental VS Wenshan	ENSBTAG00000047119	0.471351704	0.039462
Simmental VS Wenshan	DDX4	0.471378734	0.04541
Simmental VS Wenshan	CPE	0.475485123	0.003685
Simmental VS Wenshan	PAQR3	0.475935688	0.049766
Simmental VS Wenshan	ENSBTAG00000011213	0.479237412	0.013859
Simmental VS Wenshan	SLC5A12	0.483174051	0.048004
Simmental VS Wenshan	PREX2	0.485316415	0.001295
Simmental VS Wenshan	UBE2I	0.493291966	0.024465
Simmental VS Wenshan	VAV3	0.49528438	0.002739
Simmental VS Wenshan	BOLL	0.495376721	0.028219
Simmental VS Wenshan	RASGRP3	0.49552744	0.035229
Simmental VS Wenshan	ENSBTAG00000031160	0.49646677	0.003668
Simmental VS Wenshan	NOS2	0.501433196	0.001728
Simmental VS Wenshan	PDE1A	0.506340496	0.000261
Simmental VS Wenshan	URGCP	0.50646591	0.031088
Simmental VS Wenshan	ENSBTAG00000006859	0.507932475	0.009644
Simmental VS Wenshan	CCDC102A	0.512863312	0.022673
Simmental VS Wenshan	ENSBTAG00000046776	0.513424093	0.007255
Simmental VS Wenshan	C7orf72	0.517010981	0.005065
Simmental VS Wenshan	COL3A1	0.518054337	0.024375
Simmental VS Wenshan	IQUB	0.518120368	0.020697
Simmental VS Wenshan	VPS4B	0.523067589	0.012693
Simmental VS Wenshan	AK9	0.523487848	0.029765
Simmental VS Wenshan	EIF3H	0.524302047	0.034412
Simmental VS Wenshan	RBL2	0.525075144	0.022058
Simmental VS Wenshan	ENSBTAG00000047411	0.52808634	0.008191
Simmental VS Wenshan	ACTR2	0.530264306	0.049218
Simmental VS Wenshan	PTX4	0.532879238	0.024465
Simmental VS Wenshan	ANKAR	0.533317221	0.031545
Simmental VS Wenshan	TGIF2	0.537534999	0.036766
Simmental VS Wenshan	ENSBTAG00000047962	0.541922532	0.016078
Simmental VS Wenshan	SLC9B2	0.543370804	0.005958
Simmental VS Wenshan	MTIF2	0.544189861	0.043548
Simmental VS Wenshan	FSIP2	0.54610608	0.037953
Simmental VS Wenshan	ENSBTAG00000039523	0.547557735	0.037521
Simmental VS Wenshan	ENSBTAG00000047749	0.548050145	0.028347
Simmental VS Wenshan	IL12RB2	0.548165763	0.011921
Simmental VS Wenshan	ECT2L	0.548525093	0.009848

Simmental VS Wenshan	ASCC3	0.549463631	3.14E-05
Simmental VS Wenshan	LGI2	0.550858219	0.030273
Simmental VS Wenshan	TRMT11	0.553228258	0.026527
Simmental VS Wenshan	IGFBPL1	0.554289205	0.033895
Simmental VS Wenshan	HEATR1	0.554614395	0.017051
Simmental VS Wenshan	MTMR6	0.555326401	0.029581
Simmental VS Wenshan	PSMF1	0.555885675	0.043049
Simmental VS Wenshan	ENSBTAG00000012421	0.559334622	0.030435
Simmental VS Wenshan	OXR1	0.562163134	0.025668
Simmental VS Wenshan	ALCAM	0.564890849	0.017706
Simmental VS Wenshan	F7	0.564935873	0.01364
Simmental VS Wenshan	AKAP2	0.566071401	0.038775
Simmental VS Wenshan	TXK	0.567923507	0.029647
Simmental VS Wenshan	C17orf80	0.573212355	0.021109
Simmental VS Wenshan	MCRS1	0.57330081	0.025357
Simmental VS Wenshan	ENSBTAG00000000886	0.576874497	0.013724
Simmental VS Wenshan	CUX1	0.578707135	0.000315
Simmental VS Wenshan	NIPA1	0.580706827	0.033684
Simmental VS Wenshan	CENPN	0.582251415	0.037143
Simmental VS Wenshan	ZNF782	0.582659165	0.007582
Simmental VS Wenshan	UQCC1	0.583622729	0.014771
Simmental VS Wenshan	MAGI3	0.585247791	0.000543
Simmental VS Wenshan	GTF2H4	0.585830534	0.023132
Simmental VS Wenshan	ENSBTAG00000012861	0.586247071	0.007492
Simmental VS Wenshan	KCTD9	0.589436498	0.015168
Simmental VS Wenshan	ENSBTAG00000038660	0.591869586	0.0307
Simmental VS Wenshan	ERC2	0.596246478	3.35E-05
Simmental VS Wenshan	EIF3A	0.597216583	0.02925
Simmental VS Wenshan	SCNN1A	0.603034409	3.52E-05
Simmental VS Wenshan	IPO5	0.603129549	0.011423
Simmental VS Wenshan	MPP6	0.604609726	0.026124
Simmental VS Wenshan	DDB2	0.612589734	0.04072
Simmental VS Wenshan	ENSBTAG00000008852	0.619865588	0.032753
Simmental VS Wenshan	ITIH1	0.626537306	0.032247
Simmental VS Wenshan	CCDC158	0.632428396	0.006627
Simmental VS Wenshan	ENSBTAG00000019226	0.633403634	0.023485
Simmental VS Wenshan	GRXCR1	0.633832705	8.88E-06
Simmental VS Wenshan	FOXN4	0.639865637	4.34E-06
Simmental VS Wenshan	PDCL	0.644209249	0.045932
Simmental VS Wenshan	CENPF	0.647632263	0.00476
Simmental VS Wenshan	HTRA4	0.654142614	0.037098
Simmental VS Wenshan	CNTNAP5	0.654727806	0.000159
Simmental VS Wenshan	PTGR1	0.655613541	0.03725

Simmental VS Wenshan	UCK1	0.656357535	0.00277
Simmental VS Wenshan	AGAP2	0.66230376	0.017309
Simmental VS Wenshan	ENSBTAG00000034939	0.676596026	0.037509
Simmental VS Wenshan	C16orf86	0.677762189	0.047193
Simmental VS Wenshan	IMPACT	0.677863087	0.025217
Simmental VS Wenshan	RAB37	0.680293012	9.20E-05
Simmental VS Wenshan	TRIM40	0.68095359	0.028111
Simmental VS Wenshan	THOC3	0.681391409	0.000271
Simmental VS Wenshan	G6PC3	0.681761595	0.038254
Simmental VS Wenshan	FN3K	0.681949724	0.026386
Simmental VS Wenshan	ENSBTAG00000039256	0.687375822	4.60E-05
Simmental VS Wenshan	CAMK1	0.694026678	0.045798
Simmental VS Wenshan	NHLRC2	0.696167638	0.010673
Simmental VS Wenshan	DUS1L	0.697222967	0.014834
Simmental VS Wenshan	WDR3	0.703567575	0.030058
Simmental VS Wenshan	TRDMT1	0.707107318	0.015673
Simmental VS Wenshan	SLC34A3	0.708593632	0.000917
Simmental VS Wenshan	TBCEL	0.712297355	0.009831
Simmental VS Wenshan	AMHR2	0.712912818	0.045439
Simmental VS Wenshan	ENSBTAG00000033545	0.713730369	4.30E-05
Simmental VS Wenshan	NCOA4	0.71447903	0.018938
Simmental VS Wenshan	TRIM33	0.715025775	0.000108
Simmental VS Wenshan	CENPK	0.721011561	0.027803
Simmental VS Wenshan	ENSBTAG00000024632	0.733650239	0.00025
Simmental VS Wenshan	ENSBTAG00000018082	0.734798011	0.008808
Simmental VS Wenshan	JSRP1	0.737287688	0.026091
Simmental VS Wenshan	ENSBTAG00000037453	0.738170529	0.038459
Simmental VS Wenshan	RPLP0	0.742827652	0.034747
Simmental VS Wenshan	C10orf82	0.747588364	0.02079
Simmental VS Wenshan	ENSBTAG00000015648	0.753504807	0.010907
Simmental VS Wenshan	FAM186A	0.755803525	0.020915
Simmental VS Wenshan	FHL1	0.759701756	0.016971
Simmental VS Wenshan	SLC51A	0.764027183	0.035557
Simmental VS Wenshan	SDHC	0.77103709	0.013507
Simmental VS Wenshan	SNAI1	0.772795671	0.031713
Simmental VS Wenshan	RFC1	0.780088664	0.026735
Simmental VS Wenshan	PAK2	0.781886197	0.032332
Simmental VS Wenshan	SNX1	0.781960396	0.014281
Simmental VS Wenshan	ENSBTAG00000047125	0.782601027	0.015345
Simmental VS Wenshan	ENSBTAG00000035012	0.783512558	1.20E-06
Simmental VS Wenshan	ENSBTAG00000024490	0.783725112	0.017759
Simmental VS Wenshan	ANKEF1	0.787366623	0.034646
Simmental VS Wenshan	WFDC11	0.793889172	0.015965

Simmental VS Wenshan	RAET1G	0.796050278	1.29E-09
Simmental VS Wenshan	STX17	0.797517428	0.026377
Simmental VS Wenshan	ENSBTAG00000008570	0.800287774	0.036028
Simmental VS Wenshan	GPR137C	0.810321094	0.001593
Simmental VS Wenshan	MSRB2	0.811713956	0.001608
Simmental VS Wenshan	TMEM165	0.817299659	0.037178
Simmental VS Wenshan	KLF3	0.817832367	0.018581
Simmental VS Wenshan	ENSBTAG00000039425	0.833165898	0.048045
Simmental VS Wenshan	EIF4A3	0.833593011	0.008858
Simmental VS Wenshan	ENSBTAG00000000930	0.836895335	0.033046
Simmental VS Wenshan	SERPINA4	0.83992978	0.023309
Simmental VS Wenshan	C4orf32	0.845267561	0.033671
Simmental VS Wenshan	ENSBTAG00000001126	0.853616688	0.006711
Simmental VS Wenshan	ENSBTAG00000038031	0.872593792	0.044947
Simmental VS Wenshan	ZNF729	0.873721013	1.59E-05
Simmental VS Wenshan	KIAA1524	0.874732859	0.021675
Simmental VS Wenshan	FRA10AC1	0.889595931	0.018265
Simmental VS Wenshan	TARBP2	0.890097736	0.011612
Simmental VS Wenshan	POLD3	0.902410511	0.009227
Simmental VS Wenshan	UNC80	0.915994635	2.74E-05
Simmental VS Wenshan	ENSBTAG00000035736	0.933640503	0.039773
Simmental VS Wenshan	CYB561	0.934890474	0.046794
Simmental VS Wenshan	CMPK2	0.939792719	0.011354
Simmental VS Wenshan	ENSBTAG00000047324	0.947943852	0.015243
Simmental VS Wenshan	ATP6V1E2	0.947947397	0.032861
Simmental VS Wenshan	FIS1	0.950004572	0.008187
Simmental VS Wenshan	DNASE2B	0.951532824	0.015058
Simmental VS Wenshan	FABP9	0.957251571	0.033731
Simmental VS Wenshan	HSP90B1	0.961031469	0.024894
Simmental VS Wenshan	IL36G	0.96574721	0.047003
Simmental VS Wenshan	ENSBTAG00000014136	0.974197564	0.013022
Simmental VS Wenshan	ENSBTAG00000047226	0.976059501	0.024168
Simmental VS Wenshan	ZNF566	0.99693601	0.030008
Simmental VS Wenshan	ENSBTAG00000023535	1.000655052	0.020362
Simmental VS Wenshan	NCOA7	1.003167937	0.025851
Simmental VS Wenshan	DKKL1	1.01992529	0.0358
Simmental VS Wenshan	HBEGF	1.023597993	0.007827
Simmental VS Wenshan	TTPA	1.04636622	0.029852
Simmental VS Wenshan	Leptin	1.046769107	0.027409
Simmental VS Wenshan	HAPLN2	1.048262805	0.022374
Simmental VS Wenshan	TMEM220	1.056204518	0.012333
Simmental VS Wenshan	ENSBTAG00000002416	1.059423998	0.003761
Simmental VS Wenshan	TSPAN3	1.059560247	0.045283

Simmental VS Wenshan	SRP72	1.059626262	0.029313
Simmental VS Wenshan	ENSBTAG00000038112	1.061927375	4.68E-05
Simmental VS Wenshan	ENSBTAG00000048114	1.069684334	0.020951
Simmental VS Wenshan	SFT2D2	1.070189413	0.017162
Simmental VS Wenshan	GID8	1.072910408	0.005349
Simmental VS Wenshan	ENSBTAG00000013685	1.077544814	0.04391
Simmental VS Wenshan	LTA4H	1.082254916	0.035176
Simmental VS Wenshan	CLDN20	1.088190654	0.007058
Simmental VS Wenshan	STEAP4	1.096426516	0.028105
Simmental VS Wenshan	STX19	1.098362425	0.020253
Simmental VS Wenshan	MAP3K19	1.098777918	0.008541
Simmental VS Wenshan	STIL	1.103306487	0.001539
Simmental VS Wenshan	TPM1	1.103806032	0.029111
Simmental VS Wenshan	MROH6	1.108187414	0.019146
Simmental VS Wenshan	POLR2L	1.11679863	0.044502
Simmental VS Wenshan	GPHA2	1.126845713	0.023201
Simmental VS Wenshan	ENSBTAG00000037490	1.131136958	0.025352
Simmental VS Wenshan	ENSBTAG00000038261	1.139312913	0.004049
Simmental VS Wenshan	CISD2	1.145316188	0.021497
Simmental VS Wenshan	ENSBTAG00000036061	1.154032606	0.004782
Simmental VS Wenshan	GH	1.160881812	0.034635
Simmental VS Wenshan	GPR4	1.189843003	0.019039
Simmental VS Wenshan	TSSK3	1.192933826	0.019512
Simmental VS Wenshan	ENSBTAG00000047902	1.200439706	2.67E-05
Simmental VS Wenshan	PPP1CB	1.215798411	0.028115
Simmental VS Wenshan	WSB1	1.216992981	0.008996
Simmental VS Wenshan	G6PC2	1.220860754	0.028634
Simmental VS Wenshan	ENSBTAG00000031186	1.229393109	0.022078
Simmental VS Wenshan	ENSBTAG00000038890	1.243547425	0.014668
Simmental VS Wenshan	PTRH1	1.245787651	0.010204
Simmental VS Wenshan	IL11RA	1.253912478	0.016912
Simmental VS Wenshan	ERAS	1.260969759	0.036698
Simmental VS Wenshan	ZFP1	1.274394978	0.019412
Simmental VS Wenshan	ENSBTAG00000007349	1.280278452	0.045763
Simmental VS Wenshan	DNTTIP2	1.292346937	0.008277
Simmental VS Wenshan	FOXD2	1.311700019	0.010994
Simmental VS Wenshan	SULT1B1	1.323083877	0.007263
Simmental VS Wenshan	ENSBTAG00000034657	1.326793039	0.006499
Simmental VS Wenshan	ENSBTAG00000030951	1.332971057	0.009165
Simmental VS Wenshan	ENSBTAG00000046281	1.334600273	0.040551
Simmental VS Wenshan	PRLHR	1.360341364	0.010147
Simmental VS Wenshan	JMJD4	1.384089478	0.001877
Simmental VS Wenshan	GNPDA1	1.446214615	0.00177

Simmental VS Wenshan	CHAC2	1.490226465	0.011237
Simmental VS Wenshan	ENSBTAG00000026792	1.51627815	0.001322
Simmental VS Wenshan	ENSBTAG00000008035	1.525624317	0.001831
Simmental VS Wenshan	ENSBTAG00000006903	1.536814379	0.020704
Simmental VS Wenshan	MRPL52	1.540426823	0.017207
Simmental VS Wenshan	KCNS2	1.55797024	0.007169
Simmental VS Wenshan	ENSBTAG00000038222	1.563558168	1.68E-06
Simmental VS Wenshan	ENSBTAG00000047836	1.579790822	0.017178
Simmental VS Wenshan	ENSBTAG00000039021	1.590667666	0.007587
Simmental VS Wenshan	CCNB1IP1	1.608502984	0.019234
Simmental VS Wenshan	ENSBTAG00000038500	1.626481204	0.000963
Simmental VS Wenshan	ENSBTAG00000047652	1.653398157	0.035172
Simmental VS Wenshan	HEBP2	1.658831585	0.014283
Simmental VS Wenshan	HN1	1.65899763	0.005773
Simmental VS Wenshan	VSIG2	1.675266478	0.001879
Simmental VS Wenshan	HSPA12A	1.693595294	0.022851
Simmental VS Wenshan	ALDH1B1	1.706466626	0.010177
Simmental VS Wenshan	VNN1	1.706510827	0.006445
Simmental VS Wenshan	OXA1L	1.714267021	0.001457
Simmental VS Wenshan	AWAT1	1.73394269	0.009977
Simmental VS Wenshan	ENSBTAG00000024245	1.751293985	0.009835
Simmental VS Wenshan	RP9	1.767742483	0.001197
Simmental VS Wenshan	IFI27	1.793064287	0.022033
Simmental VS Wenshan	ENSBTAG00000047831	1.796842798	0.00528
Simmental VS Wenshan	GALR2	1.804019059	0.000299
Simmental VS Wenshan	CERS2	1.843716536	0.00216
Simmental VS Wenshan	SPINK7	1.913860914	0.015159
Simmental VS Wenshan	CH25H	1.928973207	0.002207
Simmental VS Wenshan	KIR3DL1	1.937280189	0.037252
Simmental VS Wenshan	HOXB2	2.068758725	0.009508
Simmental VS Wenshan	ENSBTAG00000040609	2.095921745	0.010426
Simmental VS Wenshan	ENSBTAG00000001836	2.163968078	1.23E-05
Simmental VS Wenshan	ENSBTAG00000039875	2.224306235	0.000231
Simmental VS Wenshan	ENSBTAG00000024272	2.253469337	0.000715
Simmental VS Wenshan	AHSP	2.357634438	0.000239
Simmental VS Wenshan	TMEM35	2.378037845	0.001884
Simmental VS Wenshan	ENSBTAG00000019579	2.398917142	0.00186
Simmental VS Wenshan	ENSBTAG00000004415	2.406300672	0.002288
Simmental VS Wenshan	RNF7	2.528762223	8.12E-05
Simmental VS Wenshan	MT3	2.727800644	0.004425
Simmental VS Wenshan	ENSBTAG00000039272	2.772126641	0.000182
Simmental VS Wenshan	ENSBTAG00000038625	3.118595886	5.65E-07
Simmental VS Wenshan	ENSBTAG00000004667	3.193066859	2.91E-05

Simmental VS Wenshan	ENSBTAG00000014529	3.508465869	7.80E-10
Simmental VS Wenshan	OR5C1	3.514978631	0.000864

TABLE 2

Sample name	Gene name	logFC	PValue
Simmental VS Yunnan Yunling	ENSBTAG00000046631	-6.264338388	3.85E-07
Simmental VS Yunnan Yunling	ENSBTAG00000000628	-2.987628423	0.001791
Simmental VS Yunnan Yunling	CPOX	-2.924551834	0.000193
Simmental VS Yunnan Yunling	ENSBTAG00000046884	-2.712614329	0.00213
Simmental VS Yunnan Yunling	ENSBTAG00000018542	-2.571620826	0.005358
Simmental VS Yunnan Yunling	ENSBTAG00000012692	-2.481252874	0.005202
Simmental VS Yunnan Yunling	LIAS	-2.133816807	0.025566
Simmental VS Yunnan Yunling	ENSBTAG00000022504	-2.132349507	0.02489
Simmental VS Yunnan Yunling	SNRNP35	-2.027247993	0.002017
Simmental VS Yunnan Yunling	ENSBTAG00000026543	-1.948701343	0.01307
Simmental VS Yunnan Yunling	LELP1	-1.915248985	0.009151
Simmental VS Yunnan Yunling	FOXA2	-1.912609628	0.009808
Simmental VS Yunnan Yunling	ENSBTAG00000047796	-1.912609501	0.009809
Simmental VS Yunnan Yunling	PCYOX1	-1.850206638	0.040449
Simmental VS Yunnan Yunling	ENSBTAG00000033979	-1.827886765	0.010307
Simmental VS Yunnan Yunling	TRBV29-1	-1.774519231	0.024579
Simmental VS Yunnan Yunling	ENSBTAG00000047828	-1.637625138	0.005167
Simmental VS Yunnan Yunling	ENSBTAG00000036082	-1.583599457	0.007227
Simmental VS Yunnan Yunling	CBLN1	-1.579209021	0.034431
Simmental VS Yunnan Yunling	PCDHA5	-1.547845104	0.002972
Simmental VS Yunnan Yunling	MTERF4	-1.541025308	0.019399
Simmental VS Yunnan Yunling	ENSBTAG00000031593	-1.535988161	0.028862
Simmental VS Yunnan Yunling	ENSBTAG00000046496	-1.515139901	0.032407
Simmental VS Yunnan Yunling	HFE	-1.500832757	0.028275
Simmental VS Yunnan Yunling	OR2W3	-1.477648616	0.023562
Simmental VS Yunnan Yunling	ENSBTAG00000023258	-1.434402823	0.021004
Simmental VS Yunnan Yunling	TRMT5	-1.362576949	0.014666
Simmental VS Yunnan Yunling	ENSBTAG00000014337	-1.35823075	0.035168
Simmental VS Yunnan Yunling	TRDC	-1.341136867	0.001722
Simmental VS Yunnan Yunling	GADD45GIP1	-1.307108873	0.021417
Simmental VS Yunnan Yunling	ENSBTAG00000047336	-1.292684209	0.010435
Simmental VS Yunnan Yunling	CALML6	-1.29216632	0.036526
Simmental VS Yunnan Yunling	NMRK1	-1.249009879	0.036351
Simmental VS Yunnan Yunling	ENSBTAG00000015309	-1.246499422	0.010137
Simmental VS Yunnan Yunling	ANAPC7	-1.22433197	0.037186
Simmental VS Yunnan Yunling	ENSBTAG00000046412	-1.211103033	0.037102

Simmental VS Yunnan Yunling	PTRH2	-1.209001142	0.010467
Simmental VS Yunnan Yunling	KLRF1	-1.176779713	0.029011
Simmental VS Yunnan Yunling	BAZ1A	-1.176678751	0.001757
Simmental VS Yunnan Yunling	CCDC106	-1.175630209	0.008769
Simmental VS Yunnan Yunling	ENSBTAG00000017316	-1.171561137	0.004696
Simmental VS Yunnan Yunling	JMJD8	-1.159199045	0.045121
Simmental VS Yunnan Yunling	ENSBTAG00000038160	-1.145968042	0.019958
Simmental VS Yunnan Yunling	TEX15	-1.138938487	0.045455
Simmental VS Yunnan Yunling	ENSBTAG00000025644	-1.135249162	0.042272
Simmental VS Yunnan Yunling	NOTO	-1.11443406	0.033305
Simmental VS Yunnan Yunling	PNPO	-1.107361168	0.046715
Simmental VS Yunnan Yunling	COX17	-1.105618168	0.043567
Simmental VS Yunnan Yunling	LZTS1	-1.097741027	0.003099
Simmental VS Yunnan Yunling	RRM2	-1.094035211	0.022458
Simmental VS Yunnan Yunling	ENSBTAG00000020376	-1.092071804	0.012258
Simmental VS Yunnan Yunling	TRIM31	-1.085762145	0.037027
Simmental VS Yunnan Yunling	EIF3E	-1.060430542	0.044688
Simmental VS Yunnan Yunling	ENSBTAG00000047218	-1.039523581	0.015259
Simmental VS Yunnan Yunling	ENSBTAG00000012208	-1.030161251	0.036042
Simmental VS Yunnan Yunling	GRM6	-1.013218158	0.009641
Simmental VS Yunnan Yunling	ELSPBP1	-1.008627816	0.022126
Simmental VS Yunnan Yunling	ACAD11	-1.007014888	0.033863
Simmental VS Yunnan Yunling	ENSBTAG00000034531	-1.004151914	0.015152
Simmental VS Yunnan Yunling	GABARAPL1	-1.000691762	0.037121
Simmental VS Yunnan Yunling	BCL7C	-0.996063805	0.041707
Simmental VS Yunnan Yunling	CISD1	-0.982497086	0.03594
Simmental VS Yunnan Yunling	LYPD8	-0.965428832	0.01975
Simmental VS Yunnan Yunling	ENSBTAG00000046914	-0.959870256	0.027333
Simmental VS Yunnan Yunling	FADS6	-0.935668428	0.027845
Simmental VS Yunnan Yunling	ENSBTAG00000045555	-0.927078315	0.015643
Simmental VS Yunnan Yunling	RAB12	-0.924420315	0.048615
Simmental VS Yunnan Yunling	ENSBTAG00000048188	-0.917706443	0.033384
Simmental VS Yunnan Yunling	ENSBTAG00000046433	-0.911406498	0.034049
Simmental VS Yunnan Yunling	SLU7	-0.909285826	0.046233
Simmental VS Yunnan Yunling	SNRPB2	-0.908756751	0.035581
Simmental VS Yunnan Yunling	ZNF576	-0.908723308	0.018865
Simmental VS Yunnan Yunling	ENSBTAG00000011789	-0.902707232	0.026974
Simmental VS Yunnan Yunling	ENSBTAG00000045514	-0.901203258	0.032309
Simmental VS Yunnan Yunling	LRRC31	-0.891855177	0.017045
Simmental VS Yunnan Yunling	CDC42	-0.886860852	0.029653
Simmental VS Yunnan Yunling	RNF103	-0.880767975	0.025825
Simmental VS Yunnan Yunling	SLC10A4	-0.862948531	0.011608
Simmental VS Yunnan Yunling	ENSBTAG00000021104	-0.862580277	0.001932

Simmental VS Yunnan Yunling	PLGRKT	-0.862139477	0.015906
Simmental VS Yunnan Yunling	CANX	-0.858716829	0.018697
Simmental VS Yunnan Yunling	ENSBTAG00000023309	-0.858432554	0.019524
Simmental VS Yunnan Yunling	ENSBTAG00000046325	-0.857627489	0.016251
Simmental VS Yunnan Yunling	NAA50	-0.855302012	0.006206
Simmental VS Yunnan Yunling	SLC35D2	-0.842410579	0.002305
Simmental VS Yunnan Yunling	ENSBTAG00000032350	-0.841996699	0.008506
Simmental VS Yunnan Yunling	RNPC3	-0.814490837	0.034995
Simmental VS Yunnan Yunling	TRMT1L	-0.806381333	0.036077
Simmental VS Yunnan Yunling	LYPD6	-0.802407224	0.007117
Simmental VS Yunnan Yunling	GLIS2	-0.793185425	0.013566
Simmental VS Yunnan Yunling	HYKK	-0.79004846	0.025652
Simmental VS Yunnan Yunling	DMC1	-0.788347981	0.010846
Simmental VS Yunnan Yunling	FAM196B	-0.787802801	0.012261
Simmental VS Yunnan Yunling	BTC	-0.776865499	0.038874
Simmental VS Yunnan Yunling	PIGK	-0.776790544	0.000736
Simmental VS Yunnan Yunling	SGTB	-0.775982712	0.015306
Simmental VS Yunnan Yunling	ENSBTAG00000001495	-0.76646855	0.040689
Simmental VS Yunnan Yunling	ENSBTAG00000038030	-0.762479249	0.000883
Simmental VS Yunnan Yunling	LRRC36	-0.759969136	0.039537
Simmental VS Yunnan Yunling	FAM204A	-0.75988475	0.018578
Simmental VS Yunnan Yunling	ENSBTAG00000038815	-0.748855259	0.004758
Simmental VS Yunnan Yunling	ENSBTAG00000039765	-0.739946941	0.027503
Simmental VS Yunnan Yunling	WRAP53	-0.736399469	0.048126
Simmental VS Yunnan Yunling	SAP18	-0.734508144	0.045896
Simmental VS Yunnan Yunling	CACNA2D1	-0.730684116	0.004791
Simmental VS Yunnan Yunling	ENSBTAG00000047181	-0.713823755	0.005923
Simmental VS Yunnan Yunling	NDUFA8	-0.696765282	0.033521
Simmental VS Yunnan Yunling	NOL8	-0.689501212	0.032795
Simmental VS Yunnan Yunling	DSG4	-0.686775816	0.028831
Simmental VS Yunnan Yunling	FDX1	-0.663624477	0.038168
Simmental VS Yunnan Yunling	WDR78	-0.660038853	0.004297
Simmental VS Yunnan Yunling	FAM126A	-0.654309347	0.042871
Simmental VS Yunnan Yunling	LRRC29	-0.652439229	0.021949
Simmental VS Yunnan Yunling	BDNF	-0.651232843	0.005872
Simmental VS Yunnan Yunling	HEPH	-0.645645627	0.024641
Simmental VS Yunnan Yunling	ENSBTAG00000003408	-0.644154096	0.009054
Simmental VS Yunnan Yunling	GLIPR1	-0.643211821	0.00985
Simmental VS Yunnan Yunling	CNTN6	-0.640372543	0.007164
Simmental VS Yunnan Yunling	SREK1IP1	-0.639685562	0.017835
Simmental VS Yunnan Yunling	BUB1B	-0.639620695	0.036695
Simmental VS Yunnan Yunling	SERPING1	-0.633897779	0.024366
Simmental VS Yunnan Yunling	ELF2	-0.629275672	0.016516

Simmental VS Yunnan Yunling	WDR11	-0.626611407	0.006927
Simmental VS Yunnan Yunling	TRAPPC13	-0.624743507	0.033686
Simmental VS Yunnan Yunling	KIAA1429	-0.623633737	0.021752
Simmental VS Yunnan Yunling	NPTN	-0.617959857	0.033282
Simmental VS Yunnan Yunling	PPP2R2A	-0.609636968	0.0189
Simmental VS Yunnan Yunling	PJA2	-0.605737144	0.027789
Simmental VS Yunnan Yunling	ATP1B1	-0.602487709	0.026513
Simmental VS Yunnan Yunling	MYBPC1	-0.598443672	0.014764
Simmental VS Yunnan Yunling	IPO8	-0.593964753	0.017302
Simmental VS Yunnan Yunling	NRG3	-0.592517233	0.012386
Simmental VS Yunnan Yunling	SEC63	-0.589422263	0.018303
Simmental VS Yunnan Yunling	DPP8	-0.588234121	0.046527
Simmental VS Yunnan Yunling	ZCCHC11	-0.587881411	0.04791
Simmental VS Yunnan Yunling	TRUB1	-0.587446034	0.021828
Simmental VS Yunnan Yunling	ORC5	-0.585327074	0.008686
Simmental VS Yunnan Yunling	C1orf174	-0.584749355	0.043282
Simmental VS Yunnan Yunling	KCTD3	-0.58148904	0.041677
Simmental VS Yunnan Yunling	PAX6	-0.581476086	0.044167
Simmental VS Yunnan Yunling	FANCL	-0.577861301	0.048335
Simmental VS Yunnan Yunling	MSH4	-0.574190279	0.031545
Simmental VS Yunnan Yunling	LIMA1	-0.572763561	0.017081
Simmental VS Yunnan Yunling	GLIPR1L1	-0.565901738	0.03564
Simmental VS Yunnan Yunling	RRM2B	-0.565679565	0.028516
Simmental VS Yunnan Yunling	NUP54	-0.564638323	0.037621
Simmental VS Yunnan Yunling	NUP153	-0.559768044	0.007413
Simmental VS Yunnan Yunling	TMEM68	-0.554666239	0.046236
Simmental VS Yunnan Yunling	ERP44	-0.548075369	0.042903
Simmental VS Yunnan Yunling	AMICA1	-0.544071006	0.01397
Simmental VS Yunnan Yunling	MTTP	-0.538723506	0.045064
Simmental VS Yunnan Yunling	CAB39	-0.537806726	0.01697
Simmental VS Yunnan Yunling	CTNNAL1	-0.532060231	0.026515
Simmental VS Yunnan Yunling	B4GALT5	-0.531115907	0.030324
Simmental VS Yunnan Yunling	SOAT1	-0.528751141	0.049684
Simmental VS Yunnan Yunling	RUFY3	-0.522771624	0.021245
Simmental VS Yunnan Yunling	GPR19	-0.518067305	0.018519
Simmental VS Yunnan Yunling	SMCR8	-0.510968153	0.028038
Simmental VS Yunnan Yunling	SSFA2	-0.509075966	0.026635
Simmental VS Yunnan Yunling	MSH2	-0.508445283	0.01816
Simmental VS Yunnan Yunling	PHC3	-0.508036362	0.008326
Simmental VS Yunnan Yunling	CUL3	-0.504468189	0.007238
Simmental VS Yunnan Yunling	AP4S1	-0.503853093	0.045992
Simmental VS Yunnan Yunling	PLOD2	-0.502020455	0.005057
Simmental VS Yunnan Yunling	C1R	-0.499610002	0.009384

Simmental VS Yunnan Yunling	GLIS3	-0.49343747	0.016799
Simmental VS Yunnan Yunling	HSDL2	-0.48546933	0.042725
Simmental VS Yunnan Yunling	PCDH17	-0.484920872	0.026321
Simmental VS Yunnan Yunling	ADAM32	-0.474015611	0.025852
Simmental VS Yunnan Yunling	MTURN	-0.473407154	0.049133
Simmental VS Yunnan Yunling	ATP2C1	-0.472786726	0.028602
Simmental VS Yunnan Yunling	LDAH	-0.471310957	0.041599
Simmental VS Yunnan Yunling	FAM184B	-0.468314617	0.045964
Simmental VS Yunnan Yunling	ENSBTAG00000033312	-0.467918457	0.028318
Simmental VS Yunnan Yunling	JAK2	-0.466032573	0.049141
Simmental VS Yunnan Yunling	HMOX2	-0.465808763	0.040317
Simmental VS Yunnan Yunling	RAB3C	-0.463232533	0.039456
Simmental VS Yunnan Yunling	RGS7BP	-0.457175696	0.042573
Simmental VS Yunnan Yunling	TBC1D7	-0.455141703	0.029403
Simmental VS Yunnan Yunling	MGAM	-0.454249487	0.027645
Simmental VS Yunnan Yunling	MORC1	-0.451720909	0.022381
Simmental VS Yunnan Yunling	PRMT3	-0.449348945	0.04114
Simmental VS Yunnan Yunling	ENTPD1	-0.448846086	0.036233
Simmental VS Yunnan Yunling	BRAF	-0.434285381	0.009385
Simmental VS Yunnan Yunling	SYT1	-0.433481844	0.010724
Simmental VS Yunnan Yunling	NCOR1	-0.4326079	0.014258
Simmental VS Yunnan Yunling	POC1B	-0.430711839	0.016401
Simmental VS Yunnan Yunling	NRCAM	-0.425295465	0.02596
Simmental VS Yunnan Yunling	BPTF	-0.420700316	0.031624
Simmental VS Yunnan Yunling	RALGAPA1	-0.420290887	0.038187
Simmental VS Yunnan Yunling	STRBP	-0.417765607	0.028656
Simmental VS Yunnan Yunling	HOMER1	-0.410612269	0.034812
Simmental VS Yunnan Yunling	COL21A1	-0.408982242	0.047182
Simmental VS Yunnan Yunling	REV3L	-0.401278583	0.022049
Simmental VS Yunnan Yunling	KCNJ3	-0.400640781	0.045965
Simmental VS Yunnan Yunling	CRY1	-0.397828212	0.046239
Simmental VS Yunnan Yunling	SMOX	-0.397765824	0.049469
Simmental VS Yunnan Yunling	BIRC6	-0.396362486	0.033254
Simmental VS Yunnan Yunling	SDHA	-0.392096647	0.036124
Simmental VS Yunnan Yunling	IL34	-0.372441267	0.049149
Simmental VS Yunnan Yunling	SASH1	-0.369109125	0.027444
Simmental VS Yunnan Yunling	CCDC109B	-0.365072841	0.045767
Simmental VS Yunnan Yunling	SAXO1	-0.360408232	0.028098
Simmental VS Yunnan Yunling	ENSBTAG00000044111	-0.360231893	0.037507
Simmental VS Yunnan Yunling	CEP128	-0.35512386	0.031715
Simmental VS Yunnan Yunling	ABCC2	-0.354294484	0.048028
Simmental VS Yunnan Yunling	HECW1	-0.349201584	0.042179
Simmental VS Yunnan Yunling	RAF1	-0.342487769	0.035847

Simmental VS Yunnan Yunling	ANK2	-0.340771445	0.047844
Simmental VS Yunnan Yunling	GTDC1	-0.338888533	0.033642
Simmental VS Yunnan Yunling	CNNM2	-0.335300295	0.039105
Simmental VS Yunnan Yunling	CHL1	-0.331910725	0.030635
Simmental VS Yunnan Yunling	MUC5B	0.295153909	0.041664
Simmental VS Yunnan Yunling	SMAD6	0.313463996	0.046003
Simmental VS Yunnan Yunling	KLHDC4	0.32162856	0.047564
Simmental VS Yunnan Yunling	CNTNAP5	0.324940298	0.042971
Simmental VS Yunnan Yunling	ESPNL	0.325652366	0.034396
Simmental VS Yunnan Yunling	STAB1	0.327465499	0.028453
Simmental VS Yunnan Yunling	ASCC3	0.330419624	0.019539
Simmental VS Yunnan Yunling	TMEM184B	0.331715142	0.039354
Simmental VS Yunnan Yunling	ENSBTAG00000022188	0.331949874	0.02309
Simmental VS Yunnan Yunling	RTEL1	0.340329891	0.038444
Simmental VS Yunnan Yunling	HDAC5	0.34187039	0.038548
Simmental VS Yunnan Yunling	MMEL1	0.344552634	0.041334
Simmental VS Yunnan Yunling	GPSM1	0.344833597	0.034111
Simmental VS Yunnan Yunling	ARHGAP1	0.350690277	0.037419
Simmental VS Yunnan Yunling	RRP12	0.35162432	0.034506
Simmental VS Yunnan Yunling	COL20A1	0.353769275	0.030008
Simmental VS Yunnan Yunling	COL11A2	0.357435429	0.022697
Simmental VS Yunnan Yunling	RECQL5	0.358991675	0.036206
Simmental VS Yunnan Yunling	CHD3	0.361687174	0.028003
Simmental VS Yunnan Yunling	FANCC	0.366427584	0.004426
Simmental VS Yunnan Yunling	UNC80	0.366753706	0.021299
Simmental VS Yunnan Yunling	MYO1G	0.368539337	0.028386
Simmental VS Yunnan Yunling	EFCC1	0.370179995	0.023164
Simmental VS Yunnan Yunling	TONSL	0.373138411	0.049944
Simmental VS Yunnan Yunling	FAM65B	0.374346193	0.047679
Simmental VS Yunnan Yunling	ENSBTAG00000047150	0.374439758	0.017461
Simmental VS Yunnan Yunling	IL2RA	0.375428203	0.042668
Simmental VS Yunnan Yunling	HK1	0.375950273	0.01865
Simmental VS Yunnan Yunling	ARVCF	0.376355255	0.024033
Simmental VS Yunnan Yunling	RUVBL1	0.377774246	0.03274
Simmental VS Yunnan Yunling	IPO9	0.383225611	0.042939
Simmental VS Yunnan Yunling	LMTK2	0.38424288	0.032271
Simmental VS Yunnan Yunling	ENSBTAG00000031517	0.384719911	0.003004
Simmental VS Yunnan Yunling	ENSBTAG00000022829	0.385834926	0.014891
Simmental VS Yunnan Yunling	ADRBK1	0.386063634	0.031842
Simmental VS Yunnan Yunling	YBX1	0.386864683	0.043698
Simmental VS Yunnan Yunling	DNMT1	0.387829692	0.019238
Simmental VS Yunnan Yunling	ZFHX2	0.390027215	0.029474
Simmental VS Yunnan Yunling	IDUA	0.391284208	0.038372

Simmental VS Yunnan Yunling	FAM118A	0.398332556	0.030011
Simmental VS Yunnan Yunling	TMEM201	0.399089546	0.030111
Simmental VS Yunnan Yunling	ADAMTS7	0.40110184	0.017272
Simmental VS Yunnan Yunling	ESRRB	0.401204253	0.014072
Simmental VS Yunnan Yunling	D2HGDH	0.401745668	0.033856
Simmental VS Yunnan Yunling	ENTPD8	0.40253761	0.041478
Simmental VS Yunnan Yunling	NDOR1	0.402715365	0.045176
Simmental VS Yunnan Yunling	TTLL9	0.403459982	0.035892
Simmental VS Yunnan Yunling	ARFRP1	0.403616306	0.046556
Simmental VS Yunnan Yunling	SLC12A7	0.405005371	0.011043
Simmental VS Yunnan Yunling	C21orf2	0.405310175	0.039652
Simmental VS Yunnan Yunling	WDR90	0.405998113	0.032974
Simmental VS Yunnan Yunling	DCHS1	0.409212654	0.022336
Simmental VS Yunnan Yunling	BAIAP2	0.409686036	0.010036
Simmental VS Yunnan Yunling	UVSSA	0.409843479	0.043052
Simmental VS Yunnan Yunling	TCOF1	0.411324154	0.008788
Simmental VS Yunnan Yunling	MBTPS1	0.411439836	0.03134
Simmental VS Yunnan Yunling	SLC25A29	0.411657695	0.016928
Simmental VS Yunnan Yunling	CTBP1	0.413534249	0.013569
Simmental VS Yunnan Yunling	PLEKHG5	0.417297133	0.028605
Simmental VS Yunnan Yunling	SREBF1	0.41753078	0.03818
Simmental VS Yunnan Yunling	ARHGEF12	0.420372175	0.027595
Simmental VS Yunnan Yunling	ENSBTAG00000048051	0.425466097	0.033849
Simmental VS Yunnan Yunling	SPDEF	0.425898817	0.01338
Simmental VS Yunnan Yunling	PPP2R1A	0.434178443	0.027569
Simmental VS Yunnan Yunling	CIC	0.435306045	0.028974
Simmental VS Yunnan Yunling	PTPN7	0.436306994	0.014415
Simmental VS Yunnan Yunling	ATG4B	0.436830041	0.01352
Simmental VS Yunnan Yunling	CLCN7	0.43748713	0.037696
Simmental VS Yunnan Yunling	CHSY1	0.439792363	0.016867
Simmental VS Yunnan Yunling	ENSBTAG00000039470	0.44445087	0.007686
Simmental VS Yunnan Yunling	EXOSC7	0.448656223	0.048448
Simmental VS Yunnan Yunling	OGDHL	0.458858405	0.028157
Simmental VS Yunnan Yunling	PUF60	0.462305888	0.045352
Simmental VS Yunnan Yunling	SRRD	0.466970981	0.035949
Simmental VS Yunnan Yunling	HSF1	0.468802135	0.012331
Simmental VS Yunnan Yunling	RAB20	0.469259481	0.021334
Simmental VS Yunnan Yunling	MTMR10	0.47644843	0.036277
Simmental VS Yunnan Yunling	CTSZ	0.479892769	0.042783
Simmental VS Yunnan Yunling	ABCB11	0.485482168	0.031304
Simmental VS Yunnan Yunling	CYHR1	0.488086969	0.022826
Simmental VS Yunnan Yunling	ZNF469	0.489525936	0.007213
Simmental VS Yunnan Yunling	ALDH16A1	0.492447832	0.032204

Simmental VS Yunnan Yunling	GRXCR1	0.49288	0.005424
Simmental VS Yunnan Yunling	CXCL12	0.502932781	0.047379
Simmental VS Yunnan Yunling	BPI	0.503841714	0.040609
Simmental VS Yunnan Yunling	SIVA1	0.506627025	0.033053
Simmental VS Yunnan Yunling	LPO	0.50708751	0.007151
Simmental VS Yunnan Yunling	RASGRP2	0.509070683	0.036327
Simmental VS Yunnan Yunling	ENSBTAG00000004291	0.510577577	0.021957
Simmental VS Yunnan Yunling	ATG16L2	0.512807966	0.016737
Simmental VS Yunnan Yunling	CSRP1	0.512985103	0.003063
Simmental VS Yunnan Yunling	NR2F6	0.514792698	0.037081
Simmental VS Yunnan Yunling	NECAP2	0.515404065	0.042433
Simmental VS Yunnan Yunling	STRC	0.52273534	0.026817
Simmental VS Yunnan Yunling	CPSF3L	0.527209369	0.042667
Simmental VS Yunnan Yunling	TTYH3	0.530387285	0.004598
Simmental VS Yunnan Yunling	C9orf117	0.530393349	0.040251
Simmental VS Yunnan Yunling	CAMKV	0.531923433	0.045257
Simmental VS Yunnan Yunling	NEURL3	0.537037862	0.045602
Simmental VS Yunnan Yunling	PPP2R5B	0.537380304	0.035167
Simmental VS Yunnan Yunling	TPD52L2	0.539474908	0.026698
Simmental VS Yunnan Yunling	ACTL6B	0.541076862	0.031814
Simmental VS Yunnan Yunling	SNRNP70	0.547078906	0.01139
Simmental VS Yunnan Yunling	RAET1G	0.547762641	0.001962
Simmental VS Yunnan Yunling	DGKZ	0.554677123	0.009053
Simmental VS Yunnan Yunling	GHRL	0.557227819	0.036781
Simmental VS Yunnan Yunling	SLC22A12	0.558960927	0.034262
Simmental VS Yunnan Yunling	CDH15	0.560445183	0.003185
Simmental VS Yunnan Yunling	IGFBPL1	0.561762419	0.036889
Simmental VS Yunnan Yunling	ENSBTAG00000039892	0.561967268	0.023361
Simmental VS Yunnan Yunling	ENSBTAG00000008612	0.563615761	0.017103
Simmental VS Yunnan Yunling	MAP4K2	0.566393631	0.015164
Simmental VS Yunnan Yunling	DTYMK	0.571516097	0.009104
Simmental VS Yunnan Yunling	ORAOV1	0.571775748	0.038376
Simmental VS Yunnan Yunling	ENSBTAG00000039132	0.573088224	0.03442
Simmental VS Yunnan Yunling	NAPG	0.573150295	0.017279
Simmental VS Yunnan Yunling	SLC4A7	0.57456148	0.000295
Simmental VS Yunnan Yunling	ENSBTAG00000026944	0.583233088	0.01574
Simmental VS Yunnan Yunling	PTGES	0.585800005	0.036812
Simmental VS Yunnan Yunling	FAM3A	0.596837502	0.049688
Simmental VS Yunnan Yunling	ENSBTAG00000019348	0.603196128	0.024661
Simmental VS Yunnan Yunling	SLC34A3	0.606291941	0.007911
Simmental VS Yunnan Yunling	RBPMS2	0.612259921	0.020938
Simmental VS Yunnan Yunling	MFSD12	0.636489052	0.030089
Simmental VS Yunnan Yunling	TRIM7	0.638888033	0.042655

Simmental VS Yunnan Yunling	BCAR1	0.639670093	0.007654
Simmental VS Yunnan Yunling	TBKBP1	0.64581061	0.015463
Simmental VS Yunnan Yunling	MATK	0.648383528	0.03264
Simmental VS Yunnan Yunling	GPR26	0.649454614	0.000478
Simmental VS Yunnan Yunling	ENSBTAG00000018507	0.654188279	0.036576
Simmental VS Yunnan Yunling	ENSBTAG00000045746	0.654632861	3.10E-05
Simmental VS Yunnan Yunling	F7	0.662206243	0.007336
Simmental VS Yunnan Yunling	ENSBTAG00000045635	0.663133151	0.034545
Simmental VS Yunnan Yunling	NPHS1	0.677778592	0.004368
Simmental VS Yunnan Yunling	KCNC3	0.679685141	0.027641
Simmental VS Yunnan Yunling	ENSBTAG00000010158	0.684560888	2.25E-06
Simmental VS Yunnan Yunling	PKHD1L1	0.692821814	0.032078
Simmental VS Yunnan Yunling	APEH	0.694285777	0.002243
Simmental VS Yunnan Yunling	ENSBTAG00000035012	0.694578373	1.78E-05
Simmental VS Yunnan Yunling	DEXI	0.697105684	0.009996
Simmental VS Yunnan Yunling	ATP8B4	0.701335888	0.022678
Simmental VS Yunnan Yunling	DMKN	0.703672468	0.011055
Simmental VS Yunnan Yunling	MEST	0.70672612	0.047811
Simmental VS Yunnan Yunling	SNUPN	0.710086584	0.044478
Simmental VS Yunnan Yunling	PIP4K2B	0.721375928	0.011141
Simmental VS Yunnan Yunling	COX19	0.738557138	0.02152
Simmental VS Yunnan Yunling	ENSBTAG00000001777	0.748977645	0.042072
Simmental VS Yunnan Yunling	PIGO	0.750368871	0.032527
Simmental VS Yunnan Yunling	SNAI1	0.756397903	0.043504
Simmental VS Yunnan Yunling	ENSBTAG00000047299	0.766528538	0.007438
Simmental VS Yunnan Yunling	CA4	0.767989769	0.021773
Simmental VS Yunnan Yunling	LIPE	0.770611084	0.007348
Simmental VS Yunnan Yunling	ENSBTAG00000039037	0.787389743	0.000409
Simmental VS Yunnan Yunling	ENSBTAG00000048145	0.789636117	0.002721
Simmental VS Yunnan Yunling	CDC5L	0.797482626	0.018516
Simmental VS Yunnan Yunling	ENSBTAG00000038112	0.798769768	0.002761
Simmental VS Yunnan Yunling	ENSBTAG00000040298	0.804487293	0.007242
Simmental VS Yunnan Yunling	SPRY4	0.82500986	0.012449
Simmental VS Yunnan Yunling	LYL1	0.844031667	0.042177
Simmental VS Yunnan Yunling	RNF224	0.845548531	0.04649
Simmental VS Yunnan Yunling	COX6B1	0.851201458	0.033428
Simmental VS Yunnan Yunling	SLC51A	0.855109754	0.016444
Simmental VS Yunnan Yunling	PEX11B	0.855237189	0.04233
Simmental VS Yunnan Yunling	PEX10	0.864438745	0.002419
Simmental VS Yunnan Yunling	TXNL4B	0.865665338	0.017477
Simmental VS Yunnan Yunling	PTGR1	0.875669389	0.011443
Simmental VS Yunnan Yunling	ENSBTAG00000038261	0.889009304	0.024611
Simmental VS Yunnan Yunling	ENSBTAG00000046383	0.895181065	0.031191

Simmental VS Yunnan Yunling	PREB	0.9002763	0.038018
Simmental VS Yunnan Yunling	C6orf47	0.904647851	0.047918
Simmental VS Yunnan Yunling	MAP3K19	0.916252787	0.047401
Simmental VS Yunnan Yunling	ZC3H10	0.91975099	0.015584
Simmental VS Yunnan Yunling	GDI2	0.927933811	0.036795
Simmental VS Yunnan Yunling	FOS	0.939294061	0.028409
Simmental VS Yunnan Yunling	ENSBTAG00000000930	0.946718176	0.030792
Simmental VS Yunnan Yunling	WBSCR28	0.957231222	0.02011
Simmental VS Yunnan Yunling	ENSBTAG00000036061	0.963047375	0.047163
Simmental VS Yunnan Yunling	JMJD4	0.965147347	0.033702
Simmental VS Yunnan Yunling	ENSBTAG00000038222	0.966702184	0.03462
Simmental VS Yunnan Yunling	ATP6V0C	0.966904849	0.019764
Simmental VS Yunnan Yunling	ENSBTAG00000020923	0.974055438	0.019134
Simmental VS Yunnan Yunling	FBXL15	0.974597475	0.029722
Simmental VS Yunnan Yunling	ENSBTAG00000047270	0.981224911	0.044557
Simmental VS Yunnan Yunling	NR1H2	0.988881857	0.005925
Simmental VS Yunnan Yunling	APOA5	0.993189563	0.021438
Simmental VS Yunnan Yunling	B3GNT4	1.029039616	0.015723
Simmental VS Yunnan Yunling	ENSBTAG00000047902	1.042541162	0.001453
Simmental VS Yunnan Yunling	ENSBTAG00000000719	1.054656929	0.013996
Simmental VS Yunnan Yunling	ZNF793	1.055774095	0.023634
Simmental VS Yunnan Yunling	CYB561	1.061779747	0.029329
Simmental VS Yunnan Yunling	FIS1	1.068018805	0.007292
Simmental VS Yunnan Yunling	MRPL54	1.082434289	0.016846
Simmental VS Yunnan Yunling	ENSBTAG00000008303	1.082671501	0.019507
Simmental VS Yunnan Yunling	NEU4	1.100302748	0.022156
Simmental VS Yunnan Yunling	SAC3D1	1.111027421	0.026962
Simmental VS Yunnan Yunling	ODF3L2	1.124677299	0.000703
Simmental VS Yunnan Yunling	ENSBTAG00000026080	1.136342848	0.005962
Simmental VS Yunnan Yunling	STAT3	1.150494521	0.028911
Simmental VS Yunnan Yunling	RRAD	1.154592522	0.026085
Simmental VS Yunnan Yunling	TMIGD2	1.171731477	0.043373
Simmental VS Yunnan Yunling	GJA8	1.178799498	0.034184
Simmental VS Yunnan Yunling	FILIP1L	1.189713582	0.015366
Simmental VS Yunnan Yunling	B3GALT4	1.194617352	0.012593
Simmental VS Yunnan Yunling	ENSBTAG00000039256	1.199427008	8.12E-10
Simmental VS Yunnan Yunling	COMT	1.205602535	0.040771
Simmental VS Yunnan Yunling	ZNF70	1.208256106	0.000594
Simmental VS Yunnan Yunling	ENSBTAG00000040331	1.209991399	0.007609
Simmental VS Yunnan Yunling	C10H15orf43	1.216279819	0.00975
Simmental VS Yunnan Yunling	CBLN3	1.231896558	0.043221
Simmental VS Yunnan Yunling	VAMP5	1.239807684	0.008462
Simmental VS Yunnan Yunling	PIGB	1.246931455	0.0484

Simmental VS Yunnan Yunling	ENSBTAG00000040450	1.256383819	0.032904
Simmental VS Yunnan Yunling	ENSBTAG00000046595	1.29211226	0.028762
Simmental VS Yunnan Yunling	HTR1D	1.310588932	0.006506
Simmental VS Yunnan Yunling	VPS26A	1.311724464	0.014038
Simmental VS Yunnan Yunling	ENSBTAG00000004667	1.314601687	0.03375
Simmental VS Yunnan Yunling	TMEM220	1.318284931	0.008672
Simmental VS Yunnan Yunling	ENSBTAG00000017993	1.323785005	0.024534
Simmental VS Yunnan Yunling	FGG	1.336621584	0.047677
Simmental VS Yunnan Yunling	CISD2	1.358550011	0.003499
Simmental VS Yunnan Yunling	ENSBTAG00000046889	1.376267578	0.038529
Simmental VS Yunnan Yunling	CNTF	1.39294557	0.012999
Simmental VS Yunnan Yunling	ENSBTAG00000030204	1.420785137	0.007821
Simmental VS Yunnan Yunling	SPG11	1.429145786	1.71E-05
Simmental VS Yunnan Yunling	KIAA1024L	1.443811334	0.033022
Simmental VS Yunnan Yunling	ENSBTAG00000011657	1.449449797	0.04413
Simmental VS Yunnan Yunling	UQCRFS1	1.48737594	0.045111
Simmental VS Yunnan Yunling	ENSBTAG00000039021	1.492244121	0.007257
Simmental VS Yunnan Yunling	SLC25A5	1.498611847	0.020371
Simmental VS Yunnan Yunling	ENSBTAG00000036257	1.528378693	0.044762
Simmental VS Yunnan Yunling	RTP1	1.548323957	0.002299
Simmental VS Yunnan Yunling	ENSBTAG00000023289	1.567343449	0.041238
Simmental VS Yunnan Yunling	RGS4	1.571128903	0.00854
Simmental VS Yunnan Yunling	RPS26	1.610221315	0.016287
Simmental VS Yunnan Yunling	IFITM3	1.637410343	0.02498
Simmental VS Yunnan Yunling	ANKRD37	1.649554339	0.010523
Simmental VS Yunnan Yunling	ENSBTAG00000004415	1.713528876	0.038686
Simmental VS Yunnan Yunling	ATP6V0B	1.723819708	0.009521
Simmental VS Yunnan Yunling	ENSBTAG00000010597	1.763595688	0.002345
Simmental VS Yunnan Yunling	OR6N2	1.835942128	0.008365
Simmental VS Yunnan Yunling	C8G	1.932538355	0.00093
Simmental VS Yunnan Yunling	PIGV	1.953240767	0.001545
Simmental VS Yunnan Yunling	ENSBTAG00000039257	1.968353943	0.008464
Simmental VS Yunnan Yunling	ENSBTAG00000024245	2.014162553	0.015548
Simmental VS Yunnan Yunling	CAPN1	2.034124042	0.000616
Simmental VS Yunnan Yunling	KIAA0101	2.05237812	0.024964
Simmental VS Yunnan Yunling	ZNF280A	2.153090162	0.017883
Simmental VS Yunnan Yunling	ENSBTAG00000003281	2.45967719	0.015269
Simmental VS Yunnan Yunling	ENSBTAG00000046959	3.364197185	2.35E-05
